# Inference of epigenetic subnetworks by Bayesian regression with the incorporation of prior information

**DOI:** 10.1038/s41598-022-19879-x

**Published:** 2022-11-23

**Authors:** Anqi Jing, Jie Han

**Affiliations:** Department of Electrical and Computer Engineering, University of Alberta, Edmonton, T6G 1H9 Canada

**Keywords:** Cancer, Computational biology and bioinformatics, Systems biology

## Abstract

Changes in gene expression have been thought to play a crucial role in various types of cancer. With the advance of high-throughput experimental techniques, many genome-wide studies are underway to analyze underlying mechanisms that may drive the changes in gene expression. It has been observed that the change could arise from altered DNA methylation. However, the knowledge about the degree to which epigenetic changes might cause differences in gene expression in cancer is currently lacking. By considering the change of gene expression as the response of altered DNA methylation, we introduce a novel analytical framework to identify epigenetic subnetworks in which the methylation status of a set of highly correlated genes is predictive of a set of gene expression. By detecting highly correlated modules as representatives of the regulatory scenario underling the gene expression and DNA methylation, the dependency between DNA methylation and gene expression is explored by a Bayesian regression model with the incorporation of *g-prior* followed by a strategy of an optimal predictor subset selection. The subsequent network analysis indicates that the detected epigenetic subnetworks are highly biologically relevant and contain many verified epigenetic causal mechanisms. Moreover, a survival analysis indicates that they might be effective prognostic factors associated with patient survival time.

## Introduction

With the advance of high-throughput experimental techniques, a tremendous amount of genomic-wide omics data has been available, which revolutionizes the study of cancer by making it possible to discover potential biomarkers and biological mechanisms at the genome level. The classic view that cancer progression is driven by genetic changes including mutations and chromosomal abnormalities, and later on epigenetic alterations have been considered as crucial in the progression of cancer. Epigenetic alteration refers to the functionally relevant changes to the genome without changing DNA sequences^1^, including DNA methylation, histone modification, etc. Such epigenetic alterations have been investigated in numerous studies^2,3^, which revealed that they are likely to be responsible for the reduced or increased expression in DNA repair genes and be the cause of genetic instability characteristic of cancers in early cancer progression. DNA methylation is one epigenetic mechanism, which can alter gene expression by causing the stable silencing or activation of particular genes without changing DNA sequences. It remains throughout cell divisions and can be inherited by daughter cells lasting for multiple generations. It is of great interest to cancer study since it is potentially reversible and could be returned to normal function with appropriate drugs, which makes it an excellent target for anticancer therapies^4^. Moreover, the change in DNA methylation leading to an aberrant gene expression has been considered to play a crucial role not only in cellular development and differentiation but also in disease progression^1^. Many studies have been conducted for the identification of aberrant DNA methylation sites in cancer^1,5,6^, but there are fewer studies on the degree to which epigenetic changes might cause significant differences of gene expression in cancer. Thus it motivates us to discover the association between epigenetic changes and altered gene expression and investigate the varying level of DNA methylation that could drive differences in gene expression.

A recent database TCGA (The Cancer Genome Atlas)^7^ has profiled and collected multidimensional omics data at DNA, RNA, protein and epigenetic levels for hundreds of clinical tumors, making the integrative analysis of epigenetic mechanisms at the whole genome level possible^8–10^. For example, Hinoue et al.^8^ identified four DNA methylation-based subgroups of colorectal cancer exhibiting characteristic genetic and clinical features, which provided novel insights regarding the role of subgroup-specific DNA hypermethylation in gene silencing. Varley et al.^9^ provided an atlas of DNA methylation across diverse and well-characterized samples and analyzed dynamic DNA methylation patterns in 82 cell lines and tissues, which discovered the role of DNA methylation in gene regulation and disease. Although the pattern of DNA methylation has been extensively investigated, how gene modules or pathways are deregulated through DNA methylation is far from understood. More specifically, approaches for simultaneously analyzing methylation and gene expression data need to be developed to discover how DNA methylation deregulates gene expression in cancer. Konno et al.^11^ has presented a mathematical method to simultaneously quantify the association between DNA methylation and transcription, and identified several candidates for conferring resistance to anti-cancer drugs in gastrointestinal cancer. Costa et al.^12^ have selected multi-omic data from TCGA comprising data of gene expression, methylation profiles, mutational patterns and clinical information, and identified the negative correlation between differentially expressed and methylated genes, which suggested that the genes are regulated by methylation alteration patterns in their promoters. Dabrowski et al.^13^ have built a method upon the Monte Carlo Feature Selection and Interdependencies Discovery (MCFS-ID) algorithm to search TCGA glioma dataset. They have discovered a set of significant gene expression profiles and DNA methylation sites, as well as their interdependencies, which was proved to be a good predictor of glioma patient’s survival. More specifically, they found that an important methylation feature (cg15072976) overlaps with the RE1 Silencing Transcription Factor (REST) binding site, intersect with the REST binding motif in human U87 glioma cells, and shows positive association with patient survival time. West et al.^14^ have proposed a method *EpiMod* to address whether differential DNA methylation is associated with a given phenotype of interest in the context of a protein interaction network. It started from constructing a weighted co-methylation network in the context of the human interactome model in which the edge weight represents the association between DNA methylation profiles in two connecting genes, and subsequently applied a local community detection algorithm (spin-glass) to identify differential methylation hotspots around differentially methylated genes by maximizing the sum of weights. They demonstrated the existence of epigenetic modules associated with phenotypes by applying the method to cancer and ageing. However, this approach was restricted to the DNA methylation data. Jiao et al.^15^ proposed a new approach *FEM* by expanding *EpiMod* by defining the edge weight as the combination of two statistical associations of co-methylation and co-expression. Encoding the two associations into edge weight allowed it to identify epigenetically deregulated modules in which genes showing coordinated differential methylation and differential expression. They identified the previously known deregulated pathway driving endometrial cancer development and an up-streamer of the well-known progesterone receptor tumour suppressor pathway. It is well acknowledged that the existence of anti-correlations between DNA methylation and gene expression, i.e., the changes in DNA methylation cause the silencing of gene expression. The method *FEM* detected the anti-correlated epigenetically correlated modules, however a recent study^9^ found the positive correlation between the two types of data that the increased methylation is associated with the higher level of gene expression. By assuming the existences of both negative and positive correlations, Ma et al.^16^ has proposed a multiple network algorithm *EMDN* by constructing differential co-expression and differential co-methylation networks respectively and subsequently identified the common modules appeared at both networks. *EMDN* can recognize both positively and negatively correlated modules. They demonstrated that the identified modules can serve as biomarkers to predict breast subtypes and estimate the survival time of patients. However, Wang et al.^17^ pointed out that only a small proportion of the alteration in DNA methylation leads to a corresponding change in gene expression at the same gene, therefore identifying gene modules restricted to the association between DNA methylation and gene expression at the same or adjacent genes may miss important links between the two changes. To overcome this limitation, they have proposed a multivariate regression framework *NsRRR* to identify relationships between any varying level of DNAmethylation and changes in expression of any genes. By considering expression levels of genes as responses of DNA methylation levels, they extracted a group of genes in which the expression level of a subset of genes could be regressed on the DNA methylation level of remaining genes.

Inspired by *NsRRR*, to further understand the relationship between gene expression and DNA methylation, we propose a novel framework to identify epigenetic subnetworks consisting of a set of genes with aberrant gene expression or aberrant DNA methylation level, in which the altered gene expression is deregulated by DNA methylation. Different from *NsRRR*^17^ which evaluated the association at the individual gene level, we extract a high level representation of regulatory scenarios underling both gene expression and DNA methylation in the form of gene modules, and then quantify the association between DNA methylation and gene expression at module level by a regression model. Since module-level analysis could increase the association signal and provide insight into the biological behaviours^18^, we expect that regression analysis at module level could provide complementary information to the analysis at single gene level^17^ and shed light on the discovery of new epigenetic mechanisms.

In this work, we propose an analytical framework for the discovery of epigenetic subnetworks in which aberrant gene expression is deregulated by DNA methylation levels. More specifically, we consider aberrant gene expression as the response of DNA methylation predictors. It starts with the discovery of predictor and response modules on a weighted differential network, and subsequently quantify the relationship between DNA methylation predictor modules and gene expression response modules via a Bayesian regression model with the incorporation of known protein-interaction priors. For each response module, the best subset of predictor variables is selected based on Bayesian information criterion (BIC). Statistical significance tests and biological relevance analysis show that the detected epigenetic subnetworks are significantly correlated with breast cancer genes and pathways and could be a starting point to uncover underlying epigenetic mechanisms in breast cancer and reveals potential therapeutic targets.

Here are the contributions of this work: (1) A novel analytical framework based on current statistical models is developed to detect an enriched set of epigenetic subnetworks by considering a set of highly correlated genes showing the pattern of differential co-expression/methylation instead of considering a single gene as a predictor or response. (2) This framework incorporates the prior biological knowledge as g-prior in a Bayesian regression model. It has detected more significantly correlated epigenetic subnetworks than alternative models without g-prior, indicating the effectiveness of encoding biological network information as g-prior in the selection of epigenetic subnetworks. (3) The network analysis for the detected epigenetic subnetworks reveals the direct causal mechanisms verified in the scientific literature. Finally, a survival analysis indicates that the derived modules might be effective prognostic factors associated with the patients’ survival time.

## Methods

### Overall framework

**Figure 1:**
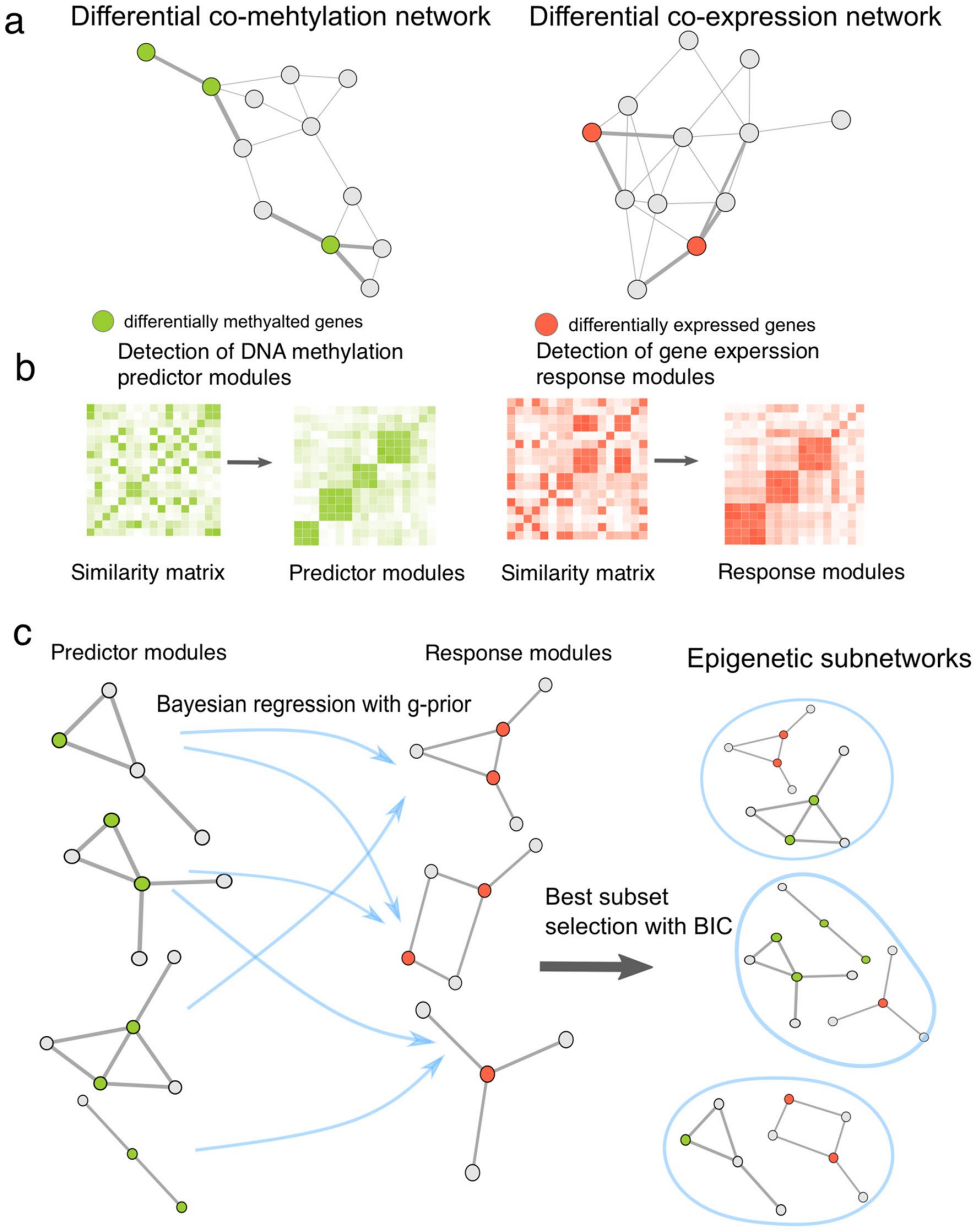
Overall framework (the figure is generated by igraph^48^ with R^33^).

As shown in Fig. 1, our method consists of three main steps. First, differential networks are constructed for gene expression and DNA methylation data in the context of the human interactome network, respectively. Edge weights are assigned according to the differential levels of gene co-expression (co-methylation) between tumor and normal conditions (Fig. 1a). Secondly, the similarity matrices are constructed by mapping the edge weights to the values of matrix elements. The gene expression and DNA methylation modules, in which genes are involved in a regulatory pattern, are discovered by the nonnegative matrix factorization to the respective similarity matrix (Fig. 1b). Since multiple DNA methylations can drive a systematic change in an expression regulatory pattern, we consider DNA methylation modules as predictors and gene expression as responses. The relationships between DNA methylation predictor modules and gene expression response modules are quantified via a Bayesian regression model with the incorporation prior biological relatedness encoded as g-prior. For each response module, the best subset of predictor variables is selected based on BIC (Fig. 1c). Finally, the response modules with its corresponding predictors compose the epigenetic subnetworks, in which the differential expression regulatory pattern results from the varying level of DNA methylation of predictors.

### Detection of predictor and response modules

#### Construction of differential networks

Since the differential expression (DE) has a cascading effect with the emergence of differential co-expression effects (DCE) due to the underlying biological network structures^19^, we combine the effects of DE and DCE in the context of a human protein-interaction network^20^ to construct the differential gene expression network. The level of DCE between each pair of interacting genes in the protein-interaction network is evaluated. Pearson correlation coefficient $\rho _t$ and $\rho _n$ are used to evaluate the correlation between two genes $X$ and $Y$ in tumor and normal conditions, respectively. Then Fisher transformations are applied to the Pearson correlation coefficients. Recall that the Fisher transformation is defined as:

1 $$\begin{aligned} F(\rho )=\frac{1}{2}\text {ln}\frac{1+\rho }{1-\rho }. \end{aligned}$$

The statistic $Z$ is defined to assess the difference in gene correlation between tumor and normal conditions:

2 $$\begin{aligned} Z=\frac{F(\rho _{t})-F(\rho _{n})}{\sqrt{\frac{1}{n_{t}-3}+\frac{1}{n_{n}-3}}},\end{aligned}$$

where $n_{t}$ and $n_{n}$ denote the number of tumor and normal samples, respectively. The absolute value of $Z$ is used as the edge weight on a pair of interacting genes in the protein interaction network.

The statistical significance on the statistic Z is evaluated:

3 $$\begin{aligned} \text {p-value} =2 \times (1-\phi (|Z|)). \end{aligned}$$

After the Benjamini–Hochberg correction, gene pairs with adjusted p-value less than 0.05 are considered to be significantly differentially co-expressed between tumor and normal conditions. To filter out irrelevant genes and reduce false positives, genes that do not show significant DCE with differentially expressed genes in the network are removed.

Analogous, differential methylation data network is constructed in the same way.

#### Detection of predictor and response modules

The constructed differential networks provide valuable information about the gene regulatory patterns, since a larger value of the edge weight indicates a higher probability that the pair of genes are involved in a gene regulatory module. To detect gene modules, two similarity matrices $A_r[a_{ij}]$ and $A_p[a_{ij}]$ are constructed, based on the differential gene expression DNA methylation network of $n$ of genes ($i, j = 1, 2,\ldots , n$). The matrix element $a_{ij}$ represents the value of the edge weight between the gene $i$ and gene $j$ in the differential network. The discovery of module structures based on the similarity matrix can be formulated as a problem of symmetric nonnegative matrix factorization (NMF). The input similarity matrix $A_r[a_{ij}]$ $(A_p[a_{ij}])$ with size $n\times n$ can be factorized into a low rank matrix *H* that encodes the latent information embedded in the original similarity matrix, i.e.

4 $$\begin{aligned} A\approx H\times H^T, \end{aligned}$$

where *H* with size $n\times k$ gives the information on module indicators, i.e., the matrix element $h_{ij}$ in *H* indicates the confidence of assigning gene $i$ to module $j$, where $i=1, 2,\ldots , n$ and $j=1, 2,\ldots , k$. The factorization of matrix $A$ can be achieved by minimizing the loss function:

5 $$\begin{aligned} min_{H\ge 0}\parallel A-H\times H^T\parallel , \textit{ subject to}\ H\ge 0. \end{aligned}$$

We solve this problem using the algorithm *SymNMF* proposed by Kuang et al.^21^, which aims to detect cluster structures where data is embedded in non-linear structure. We employ symNMF to factorize the symmetric matrices for gene expression and DNA methylation data, and exploit the module structure embedded in the graph representation. The output *H* contains the information on module memberships. Specifically, for each row $h_i$ in $H$, gene $i$ is assigned into the $k_{th}$ clusters if $h_{ik}$ is the maximum element.

#### Significance test leading to the optimal selection of predictor and response modules

The rank $K$ (i.e., the column number of matrix $H$) determines the number of modules, which is a key parameter that needs to be explored. The choice of $K$ in NMF is often an application-dependent and long-standing problem^22^. In this work, given the weighted differential network where the edge weight indicates the extent of the correlation between two genes, the task is to detect densely connected modules with high modularity. Note that not all detected modules by NMF would have above-average modularity, since it is possible that non-correlated genes would be grouped into a cluster representing isolated associations. Thus the modularity of the detected modules is evaluated by calculating module density^23^:

6 $$\begin{aligned} density(M_{ik})=\frac{\sum _{p\in M_{ik}, q\in M_{ik}}A[a_{pq}]}{|M_{ik}|\times (|M_{ik}|-1)}, \end{aligned}$$

where $M_{ik}$ indicates the $i_{th}$ module under the rank $k$ and $A$ is the similarity matrix.

A permutation test is performed to assess the statistical significance of module density by randomly generating modules with the same size as the detected module in the background differential network. This procedure is repeated 1000 times, i.e., for each detected module, 1000 random modules are generated. Under the null hypothesis, statistical significance is implied if the density of random modules is equal to or greater than that of the observed modules. Hence, the p-value is used to indicate the significance level of the density for the observed module $M_{ik}$, averaged over 1000 experiments:

7 $$\begin{aligned} p(M_{ik})=\frac{\sum _{b=1}^{b=1000}I\{density(M_{ik}^{b})\ge density(M_{ik})\}}{1000}, \end{aligned}$$

where $density(M_{ik}^{b})$ indicates the density score of the $b_{th}$ permuted module. If the adjusted p-value is less than 0.05 after the Bonferroni correction, the observed module is considered to be statistically significant.

We expect that with an appropriate value of the rank $K$, most significant modules showing local regulation patterns would be detected. A wide range value of $K$ is explored and we select the value of $K$ leading to the largest number of detected significant modules as the optimal $K$. This procedure is performed for both DNA methylation and gene expression data. The optimal values of $k_p$ and $k_r$ are respectively obtained for DNA methylation predictor modules and gene expression response modules, respectively. With the optimal values of $k_p$ and $k_r$, modules with the adjusted p-value less than 0.05 are selected as DNA methylation predictor modules and gene expression response modules, respectively.

#### Module quality measures

The module density measures whether or not genes in identified modules are densely connected. The other measure of module quality, separability score^24^, is employed to evaluate whether or not a detected module is well separated from other modules in the differential network. The separability score between two modules $M_i$ and $M_j$ is determined by the inter-module adjacency and intra-module adjacency:

8 $$\begin{aligned} separability(M_i, M_j)=1-\frac{interAdj(M_i,M_j)}{\sqrt{density(M_i)\times density(M_j)}}, \end{aligned}$$

where $density(M_i)$ and $density(M_j)$ are the intra module densities (defined in (6)) for module $M_i$ and $M_j$, respectively. The inter-module adjacency $interAdj(M_i, M_j)$ is defined as:

9 $$\begin{aligned} interAdj(M_i, M_j)=\frac{\sum _{p\in M_i}\sum _{q\in M_j}A[a_{pq}]}{n_i n_j}, \end{aligned}$$

where $A$ is the similarity matrix and $n_i$ and $n_j$ are the number of genes in $M_i$ and $M_j$, respectively. The closer the separability score is to 1, the more separated are the $M_i$ and $M_j$. The permutation test for the observed separability score is performed to obtain the significance level. The separability and density scores measure the homogeneity and separateness of the detected modules^24^. We use these two measures to validate if the modules are well detected.

### Detection of epigenetic subnetworks

In this section, we aim to identify the relationships between predictors and responses by detecting the set of predictors that best explains the variation in expression in the response module. We use the eigengene^25^ as the representative of each module in one synthetic profile, since it allows to relate the module to the clinical trait of interest in an easy way and it can also be used as a feature in more complex predictive models including the Bayesian inference model^26^. To select the best subset of predictors for each response, Bayesian linear regression model with an informative g-prior is employed to compute all possible regression models for a response module. The biological relatedness between predictors and responses is encoded as an informative g-prior to guide the search of association between predictors and responses. The best subset of predictors for each response is selected according to the criterion of Bayesian information criterion (BIC).

#### Module eigengene

We treat each modules as a single unit by constructing the representative eigengene^25^. The eigengene is defined as the first principal component based on singular value decomposition^27^. In detail, let $Y=(y_{il})$ denote the gene expression profile for a response module, where $i=1,2,\ldots ,n$ denotes the index of genes and $l=1,2,\ldots ,m$ corresponds to the tumor samples. The expression profile for each gene, i.e., each row of *Y*, is standardized to have the mean 0 and the variance 1. The singular value decomposition of $Y$ is represented as:

10 $$\begin{aligned} Y=UDV^T, \end{aligned}$$

where $U$ is an orthogonal matrix with size $n\times m$ and the columns of $U$ are referred to as the left-singular vectors. $V$ is the orthogonal matrix with size $m \times m$ and the columns of $V$ and $D$ is an $m \times m$ diagonal matrix of the singular values. The first column of $V$ is referred to as the module eigengene. Similarly, eigengenes of DNA methylation predictor modules are obtained from methylation profiles in the same way.

To evaluate if the module eigengene can represent the module profile well, we calculate the proportion of variances explained by the module eigengene^28^ as follows:

11 $$\begin{aligned} varExplained(E) = \frac{|d^1|^2}{\sum _{j}|d^j|^2}, \end{aligned}$$

where $d^1$ is the first element in the diagonal matrix $D$. The large value of $varExplained$ indicates that the module eigengene is properly generated and it can represent the profile well.

#### Bayesian regression with g-prior

For the ease of analysis, we assume that the response module is associated with a set of predictors via a linear regression model. Given a response module eigengene $Y_{i}$ and a set of predictor module eigenegenes $X_{\gamma }$, the prediction error

12 $$\begin{aligned} \varepsilon _{i\gamma }= Y_{i}-\beta _{i\gamma } \times X_{\gamma }, \end{aligned}$$

is assumed to be independent and identically distributed with mean 0 and variance $\sigma ^2$, where the parameter $\beta _{i\gamma }$ indicates the vector of regression coefficients. Assuming that the response $Y_{i}$ conditional on $X_{\gamma }$ is subject to a multivariate normal distribution:

13 $$\begin{aligned} Y_{i}|X_{\gamma },\beta _{i\gamma },\sigma ^{2} \sim Normal\bigg (\beta _{i\gamma } X_{\gamma }, \sigma _{i\gamma }^2I\bigg ), \end{aligned}$$

where $\sigma _{i\gamma }^2 I$ is a variance co-variance matrix that has error $\sigma _{i\gamma }^2$ on the diagonal and zeros for the remaining elements.

We employ Zellner’s g-prior^29^ to include the prior biological relatedness between responses and predictors. Intuitively, the g-prior controls the uncertainty in the prior belief relative to the variance of the observations around the mean, and the prior distribution of $\beta _{i\gamma }$ conditional on variance $\sigma _{i\gamma }^2$ is formulated as:

14 $$\begin{aligned} \beta _{i\gamma }|\sigma _{i\gamma }^2 \sim Normal\bigg (\beta _{i\gamma }^0, g_{i\gamma }\bigg (X_{\gamma }^TX_{\gamma }\bigg )^{-1}\sigma _{i\gamma }^2\bigg ),\end{aligned}$$

where $\beta _{i\gamma }^0$ is the initial guess of mean vector, the term $X_{\gamma }^TX_{\gamma }$ is the variance-covariance matrix that provides a prior covariance structure, and $\sigma _{i\gamma }^2$ is data-dependent covariance matrix that can be scaled by a user-defined positive factor $g_{i\gamma }$. The prior information can be integrated into the model by changing the parameter in the prior distribution, thus we can propose a prior guess of the vector of regression coefficients and encode the corresponding prior belief in $g_{i\gamma }$.

The posterior distribution of $\beta _{i\gamma }$ is given by

15 $$\begin{aligned} p\bigg (\beta _{i\gamma }|\sigma _{i\gamma }^2,X_{\gamma },Y_{i})\sim N(\frac{g_{i\gamma }}{g_{i\gamma }+1}\bigg (\frac{\beta _{i\gamma }^0}{g_{i\gamma }}+\beta _{i\gamma }^{ols}\bigg ), \frac{\sigma _{i\gamma }^{2}g_{i\gamma }}{g_{i\gamma }+1}\bigg (X_{\gamma }^TX_{\gamma })^{-1}\bigg ),\end{aligned}$$

where $\beta _{i\gamma }^{ols}=(X_{\gamma }^TX_{\gamma })^{-1}X_{\gamma }^TY_{i}$ is the OLS estimator of $\beta _{i\gamma }$, and the vector of regression coefficients $\beta _{i\gamma }$ can be estimated by the posterior mean and prior $g_{i\gamma }$:

16 $$\begin{aligned} \widetilde{\beta _{i\gamma }} = \frac{g_{i\gamma }}{1+g_{i\gamma }}+\frac{1}{1+g_{i\gamma }}\beta _{i\gamma }^{ols}.\end{aligned}$$

The posterior distribution of $\sigma _{i\gamma }^2$ follows the inverse-gamma distribution and can be estimated as

17 $$\begin{aligned} p\bigg (\sigma _{i\gamma }^2|X_{\gamma },Y_{i}\bigg )\sim IG\bigg (\frac{n_{\gamma }}{2},\frac{SSR_{i\gamma }}{2}+\frac{\bigg (\beta _{i\gamma }^0-\beta _{i\gamma }^{ols}\bigg )X^TX\frac{1}{1+g_{i\gamma }}\bigg (\beta _{i\gamma }^0-\beta _{i\gamma }^{ols}\bigg )}{2}\bigg ),\end{aligned}$$

where $n_{\gamma }$ is the number of predictors in $X_{\gamma }$ and $SSR_{i\gamma }$ is the sum of squares of the residuals of $\beta _{i\gamma }^0$.

#### Encode prior biological information as g-prior

We evaluate the biological relatedness between each response $Y_{i}$ and each predictor $X_{j}$ based on the human protein interaction network^20^. The greater number of interactions between genes in predictor and genes in response, the higher degree of biological relatedness between them. We define the biological relatedness $r_{i\gamma }$ between $X_{j}$ and $Y_{i}$ as:

18 $$\begin{aligned} r_{ij}=\frac{2E_{_{ij}}}{N{_{ij}}}, \end{aligned}$$

where $N{_{ij}}$ is the total number of genes in predictor $X_{j}$ and response module $Y_{i}$. The parameter $E{_{ij}}$ denotes the number of interactions between genes in the predictor and the response.

Then a weight parameter $\mu$ is added to control the relative influence of the prior biological relatedness in the Bayesian regression model:

19 $$\begin{aligned} g_{ij}=\mu r_{ij}. \end{aligned}$$

When $\mu =0$, we treat all predictors equally and no prior information is included in the model. The larger the value of $g_{ij}$ is, the more confident we are about that the predictor $X_j$ is associated with response $Y_i$.

Two factors, the prior coefficient vector $\beta ^0_{i\gamma }$ and the scalar $g_{i\gamma }$, need to be set. In practice, we set $\beta _{i\gamma }^0$ to be a vector with all elements having values of zeros, which reflects our prior belief in the very subtle dependence between the predictors and responses. The parameter $g_{i\gamma }$ is originally formulated as a constant to control the confidence in the coefficient $\beta ^0_{i\gamma }$. Specifically, a large value of $g_{i\gamma }$ leads the regression coefficients to be centered around $\beta ^{ols}_{i\gamma }$. On the other hand, values of $g_{i\gamma }$ with a small value leads to the solution centered around $\beta ^0_{i\gamma }$. We extend the formulation of $g_{i\gamma }$ as a vector $\mathbf {g_{i\gamma }}$ to allow for different levels of the control in the elements in $\beta ^0_{i\gamma }$. Each entry in $\mathbf {g_{i\gamma }}$ corresponds to one predictor, controlling the confidence in the prior belief relative to the variance of the observations around the mean. In this case, the vector $\mathbf {g_{i\gamma }}$ constructed for response $Y_i$ and the predictor set $X_{\gamma }$ is composed of $[g_{ix}]$, where $x$ indicates the index of predictors in the set $X\gamma$.

#### Best predictor subset selection based on BIC

Assuming that $k_p$ predictors are obtained, there are totally $2^{k_p}$ combinations of predictor variables for each response. As a first choice, we use BIC as a measure to select the best model. Recall that BIC is defined as:

20 $$\begin{aligned} BIC=-2\text {ln}({\hat{L}})+k\text {ln}(n),\end{aligned}$$

where $n$ is the number of observations, $k$ is the number of parameters estimated by the model and ${\hat{L}}$ is the maximum likelihood value of the model. The expected value of BIC is calculated as:

21 $$\begin{aligned} E\bigg [BIC_{i\gamma }\bigg ]=nE\bigg [ln(\sigma ^2_{i\gamma })\bigg ]+k_{\gamma }ln(n), \end{aligned}$$

where n is the number of samples and $k_\gamma$ is the number of predictors in the set $\gamma$. The expected value of $\ln (\sigma ^2_{i\gamma })$ is calculated as:

22 $$\begin{aligned} E\bigg [ln(\sigma _{i\gamma }^2)\bigg ]=Digamma\bigg (\frac{n}{2}\bigg )-In\bigg (\frac{SSR_{i\gamma }}{2}+\frac{\bigg (\beta _{i\gamma }^0-\beta _{i\gamma }^{ols}\bigg )G_{i\gamma }X_{\gamma }^TX_{\gamma }G_{i\gamma }\bigg (\beta _{i\gamma }^0-\beta _{i\gamma }^{ols})}{2}\bigg ), \end{aligned}$$

where $G_{i\gamma }$ is a square matrix in which diagonal elements are $\sqrt{\frac{1}{1+\mathbf {g_{i\gamma }}}}$ and the remaining elements are all zeros, and $SSR_{i\gamma }$ is the sum of squares of the residuals of the ordinary least squares $\beta _{i\gamma }^{ols}$. Given a response module, the combination of predictors with the smallest expected value of BIC would be selected.

#### Survival analysis

We hypothesized that the detected modules or subnetworks might be effective prognostic parameters that are associated with the survival time of patients. Thus, a survival analysis was performed. Since the coefficient in a Cox regression model is related to the hazard, i.e., a positive value represents a worse prognosis and a negative value indicates a positive association with survival time^30^. Thus, we devise the prognostic index scores for patients based on the coefficients in the Cox regression model of each module or subnetwork. The prognostic index score for a patient $i$ with a response or predictor module $k$ is defined as:

23 $$\begin{aligned} PI_{ki} = \beta ^{cox}_k E_{ki}, \end{aligned}$$

where $\beta ^{cox}_k$ is the Cox regression coefficient for module $k$ and $E_{ki}$ is the value of eigengene of module $k$ for patient $i$.

For a subnetwork $k$, the multivariate Cox regression is performed and the prognostic index score for the patient $i$ is defined as:

24 $$\begin{aligned} PI_{ki} = \sum _{c\in k}{\beta ^{cox}_{c}E_{ci}}, \end{aligned}$$

where $\beta ^{cox}_c$ is the Cox regression coefficient for a module variable $c$ in the subnetwork $k$ and $E_{ci}$ is the value of module eigengene$c$ for the patient $i$.

Then we divide patients into two groups based on the prognostic index scores: low-risk (the PI score $< 30{\text {th}}$ percentile of the entire PIs) and high-risk (the PI score $> 70{\text {th}}$ percentile of the entire PIs). Kaplan-Meier estimator is used to generate the survival curves for two groups, followed by the Log-rank test to the significance level on the difference of the survival time in two groups.

## Results

### Simulation study

To test if the proposed Bayesian regression model can identify true relationships between predictors and responses, we first applied the method to the simulation datasets. We simulated predefined epigenetic subnetworks consisting of a set of predictors and a response. The result of simulation study is presented to characterize the ability of our method in detecting true epigenetic subnetworks.

#### Simulation dataset

We generated three sets of studies corresponding to different strengths of association $c_r=(0.3, 0.5, 0.7)$ within response modules. In each study, we generated four predictor modules, and simulated different levels of associations from $\Phi = (0.03, 0.05, 0.1, 0.2, 0.3)$ between predictors and responses to detect the true relationships with respect to different strengths of associations. Since the structure of epigenetic subnetworks was known, the performance of the model can be evaluated by comparing the detected structure to the predefined structure.

First, we simulated four DNA methylation predictors $x_1, x_2, x_3, x_4$ corresponding to different correlation signals $c_p = (0.3, 0.5, 0.3, 0.5)$, with the same size $n \times p$, where $n$ and $p$ indicate the number of samples and variables, respectively. In practice, we set $n = 200$ and $p = 25$. Let $x_i^m$ denote the methylation level of the variable $m$ in the $i_{th}$ predictor module, which is generated as:

25 $$\begin{aligned} x_i^m\sim N(0, \Sigma _i^m), \end{aligned}$$

where $\Sigma _i^m \sim Inverse{-}Wishart(60, (1-c_i)I+c_iJ )$, $c_i$ is the association signal taken from $c_p$, $I_{p\times p}$ is the identity matrix and $J_{p\times p}$ is a matrix with all entries as 1.

We generated three response modules $y_1, y_2, y_3$ with size $n \times q$, corresponding to different levels of correlations, in order to test if different levels of correlation within responses would affect the outcome of our method. In practice, we set $n = 200$ and $q = 30$. Thus, three response modules with correlation signals $c_r = (0.3, 0.5, 0.7)$ were generated in a similar way as the predictor modules.

We further simulated subnetwork structures by assuming that specific predictors and responses contribute to the non-random associations. Let $x_j$ and $y_i$ denote the profile of the $j_{th}$ module in $x$ and the $i_{th}$ module in $y$, respectively. The dependency between response $y_i$ and a specific set of predictors is added. Thus the new profile of the $i_{th}$ response $y^S_{i}$ was obtained as follows:

26 $$\begin{aligned} y^S_{i}=y_{i}+\sum _{i\in S_{i}}{x_iA}+E, \end{aligned}$$

where $S_{i}$ indicates the set of predictors having associations with $y_i$, $A$ is a matrix of size $p\times r$ with elements carrying the association signal from $\Phi = (0.03, 0.05, 0.1, 0.2, 0.3)$, and $E$ is the random noise matrix with size $n\times q$ generated from the independent normal distribution with mean 0 and variance 1. In practice, we set $S_1$, $S_2$ and $S_3$ as $\{x_1\}$, $\{x_2\}$, $\{x_1, x_2\}$, respectively, which means that the response modules $y_1$ and $y_2$ are regulated by predictors $x_1$ and $x_2$ respectively, while $y_3$ is regulated by both $x_1$ and $x_2$. No predictor is specified for response module $y_4$.

In addition, we generated a gene-interaction network $G$ to simulate the biological relatedness between responses and predictors by using two parameters: $p_c$ is the probability of the connection between the predictor and response that belong to the same epigenetic subnetwork, and $p_{cn}$ is the probability of the connection between the predictor and response not in a a same epigenetic subnetwork. We set $p_c = 0.1$ and $p_{cn}=0.05$ such that predictor modules and response modules in the same subnetwork are relatively densely connected, whereas there are fewer links in the rest of epigenetic subnetworks.

#### Results and discussion

Three sets of dataset are simulated corresponding to different levels of correlations within response modules. We applied our method to the three datasets starting with the construction of module eigengenes. The simulated methylation profile of two predictor modules across 200 samples with correlation signal 0.3 and 0.5 were presented in Supplementary Fig. 1. An intuitive illustration of eigengene is shown by the black line in the figure and it is highly correlated with the methylation profiles in the module.

**Table 1:** Simulation results on three datasets by two methods.

(a) Result on $y_1$											
Response	True predictor	Identified predictors									
		a = 0.03		a = 0.05		a = 0.1		a = 0.2		a = 0.3	
		g-prior	No prior	g-prior	No prior	g-prior	No prior	g-prior	No prior	g-prior	No prior
$y^1_1$	$x_1$	$x_1{}^{\textstyle *}$	$x_1{}^{\textstyle *}$	$x_1{}^{\textstyle *}$	$x_1{}^{\textstyle *}$	$x_1{}^{\textstyle *}$	$x_1{}^{\textstyle *}$	$x_1{}^{\textstyle *}$	$x_1{}^{\textstyle *}$	$x_1$	$x_1{}^{\textstyle *}$
$y^1_2$	$x_2$	$x_2{}^{\textstyle *}$	$x_2{}^{\textstyle *}, x_3{}^{\textstyle *}$	$x_2{}^{\textstyle *}$	$x_2{}^{\textstyle *}$	$x_2{}^{\textstyle *}$	$x_2{}^{\textstyle *}$	$x_2{}^{\textstyle *}$	$x_2{}^{\textstyle *}$	$x_2{}^{\textstyle *}$	$x_2{}^{\textstyle *}$
$y^1_3$	$x_1,x_2$	$x_1{}^{\textstyle *}$	$x_1{}^{\textstyle *}, x_2{}^{\textstyle *}$	$x_1{}^{\textstyle *}, x_2{}^{\textstyle *}$	$x_1{}^{\textstyle *}, x_2{}^{\textstyle *}$	$x_1{}^{\textstyle *}, x_2{}^{\textstyle *}$	$x_1{}^{\textstyle *}, x_2{}^{\textstyle *}$	$x_1{}^{\textstyle *}, x_2{}^{\textstyle *}$	$x_1{}^{\textstyle *}, x_2{}^{\textstyle *}, x_4{}^{\textstyle *}$	$x_1{}^{\textstyle *}, x_2{}^{\textstyle *}$	$x_1{}^{\textstyle *}, x_2{}^{\textstyle *}$
$y^1_4$	No predictor	$x_3$	$x_3$	$x_4$	$x_4$	$x_4$	$x_4$	$x_3$	$x_3$	$x_3$	$x_3$

(b) Result on $y_2$											
Response	True predictor	Identified predictors									
		a = 0.03		a = 0.05		a = 0.1		a = 0.2		a = 0.3	
		g-prior	No prior	g-prior	No prior	g-prior	No prior	g-prior	No prior	g-prior	No prior
$y^2_1$	$x_1$	$x_1{}^{\textstyle *}$	$x_1{}^{\textstyle *}$	$x_1{}^{\textstyle *}$	$x_1{}^{\textstyle *}$	$x_1{}^{\textstyle *}$	$x_1{}^{\textstyle *}$	$x_1{}^{\textstyle *}$	$x_1{}^{\textstyle *}$	$x_1{}^{\textstyle *}$	$x_1{}^{\textstyle *}$
$y^2_2$	$x_2$	$x_2{}^{\textstyle *}$	$x_2{}^{\textstyle *}$	$x_2{}^{\textstyle *}$	$x_2{}^{\textstyle *}$	$x_2{}^{\textstyle *}$	$x_1{}^{\textstyle *}, x_2{}^{\textstyle *}$	$x_2{}^{\textstyle *}$	$x_2{}^{\textstyle *}$	$x_2{}^{\textstyle *}$	$x_1{}^{\textstyle *}, x_2{}^{\textstyle *}$
$y^2_3$	$x_1,x_2$	$x_1{}^{\textstyle *}, x_2{}^{\textstyle *}$	$x_1{}^{\textstyle *}, x_2{}^{\textstyle *}$	$x_1{}^{\textstyle *}, x_2{}^{\textstyle *}$	$x_1{}^{\textstyle *}, x_2{}^{\textstyle *}$	$x_1{}^{\textstyle *}, x_2{}^{\textstyle *}$	$x_1{}^{\textstyle *}, x_2{}^{\textstyle *}$	$x_1{}^{\textstyle *}, x_2{}^{\textstyle *}$	$x_1{}^{\textstyle *}, x_2{}^{\textstyle *}$	$x_1{}^{\textstyle *}, x_2{}^{\textstyle *}$	$x_1{}^{\textstyle *}, x_2{}^{\textstyle *}$
$y^2_4$	No predictor	$x_4$	$x_4$	$x_4$	$x_4$	$x_4$	$x_4$	$x_4$	$x_4$	$x_4$	$x_4$

(c) Result on $y_3$											
Response	True predictor	Identified predictors									
		a = 0.03		a = 0.05		a = 0.1		a = 0.2		a = 0.3	
		g-prior	No prior	g-prior	No prior	g-prior	No prior	g-prior	No prior	g-prior	No prior
$y^3_1$	$x_1$	$x_1{}^{\textstyle *}$	$x_1{}^{\textstyle *}$	$x_1{}^{\textstyle *}$	$x_1{}^{\textstyle *}$	$x_1{}^{\textstyle *}$	$x_1{}^{\textstyle *}$	$x_1{}^{\textstyle *}$	$x_1{}^{\textstyle *}$	$x_1{}^{\textstyle *}$	$x_1{}^{\textstyle *}$
$y^3_2$	$x_2$	$x_2{}^{\textstyle *}$	$x_2{}^{\textstyle *}$	$x_2{}^{\textstyle *}$	$x_2{}^{\textstyle *}$	$x_2{}^{\textstyle *}$	$x_1{}^{\textstyle *}, x_2{}^{\textstyle *}$	$x_2{}^{\textstyle *}$	$x_2{}^{\textstyle *}$	$x_2{}^{\textstyle *}$	$x_1{}^{\textstyle *}, x_2{}^{\textstyle *}$
$y^3_3$	$x_1,x_2$	$x_1{}^{\textstyle *}, x_2{}^{\textstyle *}$	$x_1{}^{\textstyle *}, x_2{}^{\textstyle *}$	$x_1{}^{\textstyle *}, x_2{}^{\textstyle *}$	$x_1{}^{\textstyle *}, x_2{}^{\textstyle *}$	$x_2{}^{\textstyle *}, x_4{}^{\textstyle *}$	$x_1{}^{\textstyle *}, x_2{}^{\textstyle *}$	$x_1{}^{\textstyle *}, x_2{}^{\textstyle *}$	$x_1{}^{\textstyle *}, x_2{}^{\textstyle *}, x_4{}^{\textstyle *}$	$x_1{}^{\textstyle *}, x_2{}^{\textstyle *}$	$x_1{}^{\textstyle *}, x_2{}^{\textstyle *}$
$y^3_4$	No predictor	$x_2$	$x_2$	$x_2$	$x_2$	$x_2$	$x_2$	$x_2$	$x_2$	$x_2$	$x_2$

a—Indicates the association signal.

*Indicates the regression coefficient is statistically significant.

**Table 2:** Breast cancer genes in detected epigenetic subnetworks.

Subnetwork	Breast cancer genes	Ratio
1	GRB7 SRC MED24 NCOA4 BCL2 PIK3CB	6/78
2	IDH1 ESR2 FOXO1 HSD17B12 PTPRJ CDKN2C SRC	7/79
3	CRK EDNRB	2/28
5	FOXM1 CLSPN MELK MMP14	4/41
6	NR3C1 PIK3CA FGF2	3/40
7	VIM ABCB1 GATA3 SFPQ ERBB4 SRC	6/69
8	CDK1 TOP2A	2/27
9	MAPK14 IKBKE SLC9A1 SRC MBIP KDM1A SERPINE1	7/94
10	FASN TWIST1 SRC	3/69
11	CSNK1A1 PTGES3 RUNX1 SRC MBIP KDM1A SERPINE1 AKT2 MAPK1 HMGB1	10/117
12	BMP6 TGM2 SRC	3/71
13	CHD4	1/51
14	CCNB1 RAD51 SRC	3/67
15	BAG3 EGFR SRC	3/70
16	CDK2 PRLR CDH1 ERBB3 TP53BP2 SRC MBIP KDM1A SERPINE1	9/92
17	CAV1	1/34
19	SOS1 PRKCZ SMYD3 ERBB2 SRC	5/68
20	SPTAN1 AHNAK ITGB1 NDRG1 ITSN1 EPAS1 MSN SRC	8/89
22	SMARCA4 MYH14 SRC	3/77
23	SMG1	1/34
24	IGF1R AKT2 PPARG SRC	4/66
25	DNMT3B ETS1	2/28
26	FN1 RBPJ FGFR1 KDM1A TGFBR2 VDR ITGB3	7/49
27	RNF41 SPARC	2/49
28	TFAP2A	1/37
29	FUBP1 IRS1 MAPK3 BTRC PIK3R1 ANK3	6/54
30	RHOA KDM5B MAP3K1 SRC MBIP KDM1A SERPINE1	7/112
31	PRKAR1A FUS MYH9 MKL1 HNRNPM SRC	6/84
32	CSK SMAD2 SOS2 TNPO1 SRC MBIP KDM1A SERPINE1	8/95
33	KDR FHL2 CD9 STAT5B PTN TP73 SRC	7/61
34	GAPDH APEX1 SRC	3/72
35	HDAC1 MUC1 HSPA5 PDIA6 RAB11FIP1 SRC MBIP KDM1A SERPINE1	9/104
36	PAK1 NOTCH1 GSN HSP90AA1 SRC MBIP KDM1A SERPINE1	8/104
38	CTNNB1 HSD17B4 ACO1 CD36 SRC	5/73
39	STUB1	1/29

For comparison, we applied the standard regression model without incorporation of prior knowledge to the simulated dataset. Results on three datasets $y_1$, $y_2$ and $y_3$ by two methods are shown in Table 1a, b and c, respectively.

**Figure 2:**
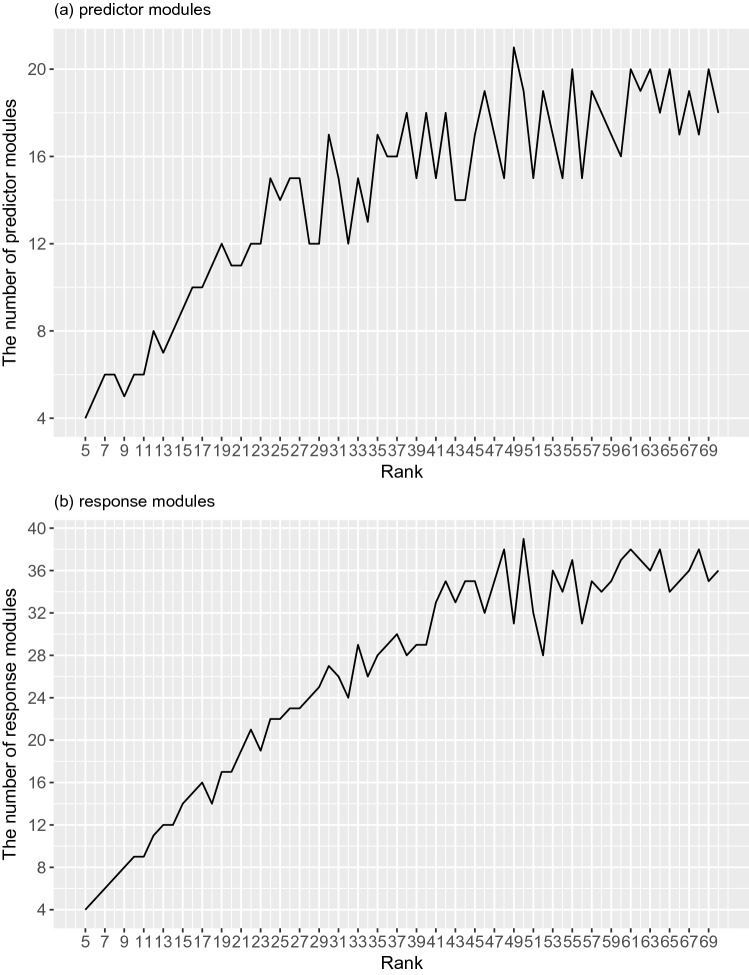
The number of predictor modules (**a**) and response modules (**b**) showing significant density score with respect to the parameter rank. The x-axis represents the candidate values for parameter *K* and y-axis represents the number of significant modules.

Table 1a shows the result of identification on the first dataset $y_1$, where ’g-prior’ indicates the result of our method with the incorporation of g-prior and ’no prior’ indicates the result of the standard regression without prior. A wide range of association strengths between responses and predictors were specified from 0.03 to 0.3. When $a = 0.03$, a very weak association was specified between the response and predictor. In the case where the single predictor is specified to the response, our method can detect almost all true relationships. When stronger associations $a = 0.05, 0.1, 0.2, 0.3$ are specified, our method identified all the true relationships on $y_1$, $y_2$ and $y_3$. However, for the method without the incorporation with g-prior, it resulted in several false positives and cannot identify true relationships as correctly as our method. For example, the false positive $x_2$ was detected by the standard model for the response $y_2^2$ and $y_2^3$, which was not specified in the relationship. Supplementary Fig. 2 shows the two examples of fitted regression models constructed by our method.

The simulation analysis demonstrated that our model can identify subnetworks correctly even in the case where a very weak association is specified, while the standard regression model without g-prior resulted in multiple false positives. The g-prior in our method worked as a modifier on the shrinkage incurred on each predictor parameter. A larger value of g-prior corresponds to a smaller shrinkage incurred on the corresponding regression coefficient, making the corresponding variable less likely to be shrunk out of the model. It is worth nothing that it only modified the degree of shrinkage of a predictor, but not the correlation between responses or the order in which the predictors are selected by the model.

### Case study

#### Dataset

We collected sample matched level-3 Illumina 450k methylation data and HiSeq RSEM gene-normalized RNA-seq data of breast cancer from TCGA^7^. We followed the strategy used by Jiao et al.^15^ to assign the methylation value to a given gene, which was introduced in “Construction of differential networks” section in detail. After data preprocessing, we generated the sample matched gene expression and DNA methylation profiles in 786 invasive ductal carcinoma tumor samples as well as 84 normal samples.

In addition, TCGA provides the corresponding clinical information including the patient status (alive or dead), the survival days (days to last follow-up or days to death). Such information was also collected to perform the survival analysis.

The information of the protein–protein interaction (PPI) was used in the inference procedure. It refers to the physical contact of high specificity between two proteins and it has been studied from multiple perspectives such as molecular dynamics, signal transduction and so on^31^. We downloaded the PPI network from the Protein Interaction Network Analysis (PINA) platform^20^, which integrates and annotates the data from six public PPI databases (MINT, IncAct, DIP, BioGRID, HPRD, and MIPS/MPact). The network consists of 166,776 edges and 16,182 nodes.

#### Discovery of predictor and response modules

Differential gene expression and DNA methylation networks were constructed by evaluating the differential co-expression and co-methylation in the PPI network. Two respective similarity matrices were generated by mapping the edge weight in the differential networks into the value of matrix elements, where an element indicates the probability that two genes may be involved in a regulatory pattern, i.e., the same module. Next, *SymNMF* was performed on these two similarity matrices to discover predictor and response modules. A wide range of candidate values from 5 to 70 for the number of modules $K$ was explored. We expected that with an appropriate value of *K*, the most number of modules showing significant high-density would be detected. Given a candidate value for *K*, density scores were calculated for detected modules. By performing the significance test, the statistical significance of the module density was evaluated. Figure 2 shows the number of the predictor and response modules showing significant density with respect to the parameter $K$. We observed that with the increase of $K$, the number of significant predictor and response modules increases to a maximum point followed by a decrease in the number of modules. The maximum number of the significant predictor and response modules were detected when $k_p$ and $k_r$ are set to 50 and 49, respectively. When $K$ exceeded the optimal value, the number of significant modules did not grow with the increase of $K$ any more. It indicated that in the case where K is greater than the optimal value, dissimilar genes were grouped into more non-correlated modules. Finally, 21 significant predictor modules and 39 significant response modules were detected with adjusted p-values less than 0.05.

#### Module quality measures

##### Density-based measure

**Figure 3:**
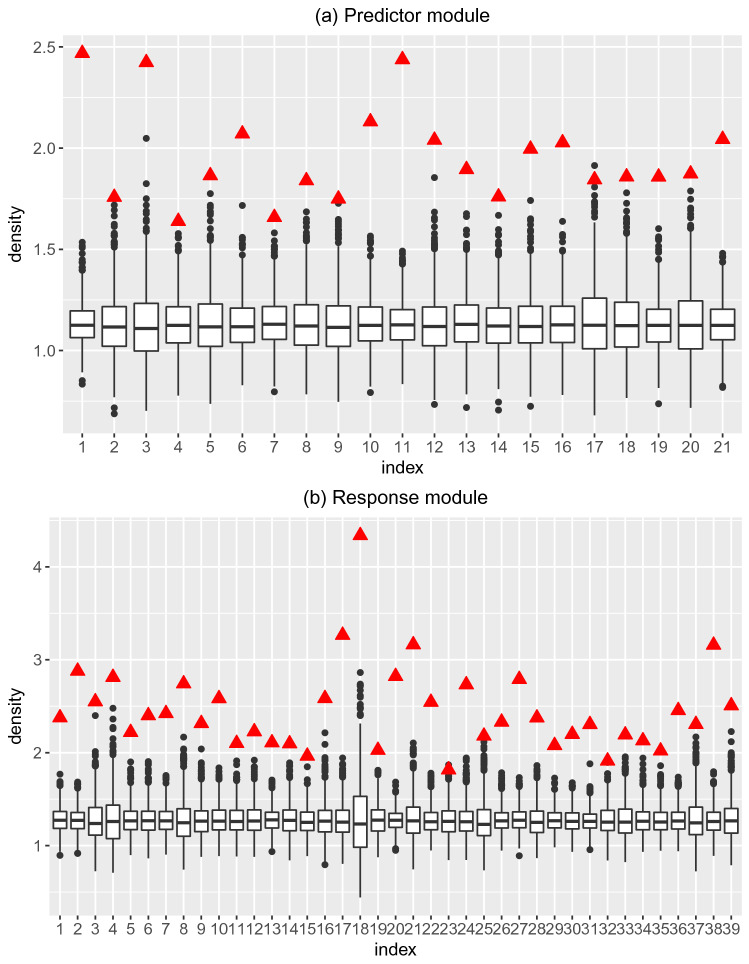
Module density scores. (**a**) The density of identified predictor modules. The red triangles represent observed density scores for predictor modules and boxplots represent the corresponding density scores of 1000 randomly generated modules. (**b**) The density of identified response modules. The red triangles represent observed density scores for response modules and boxplots represent the corresponding density scores of 1000 randomly generated modules.

As we discussed, we employed the module density to select the significant modules which remain densely connected in the differential networks. Significance levels of density statistics were measured by a permutation test. We showed the result of the permutation test in Fig. 3, where we presented the density of observed modules as well as the distribution of the densities of 1000 randomly modules. From Fig. 3, we can see that the density scores for detected modules are significantly higher than random scores.

##### Separability-based measure

Next we evaluated the separability of identified modules to test if modules remain distinct from others. Separability scores and corresponding p-value were calculated to evaluate the significance levels of the separability for each pair of identified modules. By setting the threshold of p-value as 0.05, we observed that all pairs of predictors and responses are of significant separability. The p-values of separability scores for both predictor and response modules were attached in Supplementary Appendix A. Two heatmaps (Supplementary Fig. 3, generated by ggplot2^32^ with R^33^) shows the separability and the density scores between each pair of modules, where the off-diagonal blocks represent the separability scores and the diagonal blocks represent module density. Evaluations on the density and separability revealed that the modules are well defined and genes within a module remain densely connected as well as distinct from other modules.

**Figure 4:**
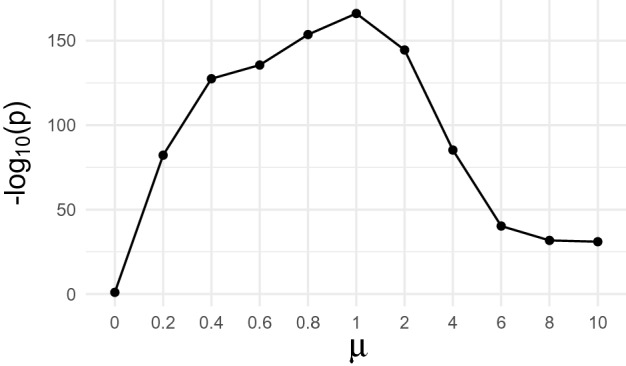
Effect of different weights on performance. It shows the negative logarithm of Fisher’s meta analyzed p-value with different weight values.

##### Other measures

We calculated $varExplained$, the proportion of the variance explained by module eigengenes, to check if the module profile is well represented by the eigengene. Supplementary Fig. 4 shows the boxplots of $varExplained$ for predictor and response modules. The median values of $varExplained$ for predictors and responses were 0.82 and 0.80, respectively, which indicated the eigengene can represent a large proportion of variance of the module profile.

**Figure 5:**
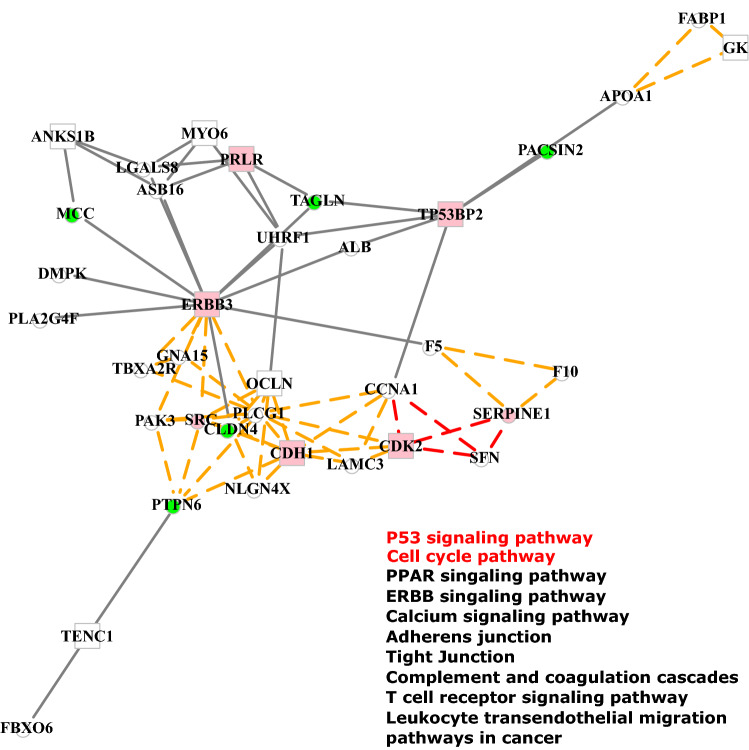
Network analysis of the detected subnetwork 16 in breast cancer. Genes that acted as predictors are represented by circles and responses are represented by squares. Pink nodes denote breast cancer driver genes and green nodes denote cancer genes. A grey line indicates that a Pearson correlation coefficient between a predictor and a response is larger than 0.03. Genes enriched in KEGG breast cancer pathways^49^ were connected by red dash lines and the yellow dash lines denote other KEGG pathways. The names for KEGG pathways are shown at the right bottom corner, where the red text indicates the breast cancer specific pathways.

In addition, we evaluated if the detected predictor and response modules are correlated with patient survival time. We selected the right-censoring tumor samples, i.e., patients with known death time, to measure the correlation between the module eigengene and the survival time of patients. The Pearson correlation coefficients and the corresponding significance levels by the permutation with z-test were calculated. The modules with p-value less than 0.05 are considered to be associated with the patient survival time. We found that 13 out of 39 response modules are significantly correlated with the patient survival time, while no significant correlations between predictors and survival time were found. Supplementary Figure 5 showed scatterplots between eigengenes and the patient survival time for the 13 response modules.

### Discovery of epigenetic subnetworks

The Pearson correlation between the profiles of DNA methylation and gene expression within each detected subnetwork are calculated to evaluate the performance. The p-value on the correlation coefficients after adjustment by a permutation test was obtained for each detected subnetwork. The Fisher’s meta analyzed p-value was obtained by combining the set of p-values for all subnetworks into one meta p-value using Fisher’s combined probability test to evaluate the overall performance. The weight parameter $\mu$ on g-prior was set to control the relative influence of prior biological relatedness to the discovery of epigenetic subnetwork. We measured the sensitivity of our method to the weight $\mu$ from a wide range of candidates $[0, 0.2, 0.4, 0.6, 0.8, 1, 2, 4, 6, 8, 10]$. Different sets of epigenetic subnetworks were detected and the Fisher’s meta analyzed p-values were obtained with respect to different values of the parameter $\mu$.

Figure 4 shows the negative logarithm of Fisher’s meta-analyzed p-value for each $\mu$. When $\mu =0$, no prior information was incorporated. As the value of $\mu$ increases, the performance increases to a certain point then followed by a decrease. The best performance was obtained when $\mu =1$, therefore we selected the value of 1 leading to the most significant correlation within subnetworks as the optimal weight value on the prior. We noticed that in the case where $\mu =0$, not all detected subnetworks show a significant correlation, which indicated that the incorporation with g-prior contributed to the discovery of significant epigenetic subnetworks.

**Table 3:** Regression results.

Response	Predictor	Coefficient	p-value	Response	Predictor	Coefficient	p-value
**y1**	x3	− 1.04	2.09E−02	**y20**	x2	− 0.69	1.41E−58
	x8	1.00	6.51E−22		x14	− 0.27	5.12E−12
**y20**	x15	− 0.63	7.47E−18	y21	x5	− 0.04	0.313
	x19	− 0.27	1.86E−04	**y22**	x3	− 0.10	3.98E−23
**y3**	x13	− 0.24	2.51E−11		x8	− 1.00	1.93E−21
**y4**	x7	− 0.38	1.33E−28	**y23**	x15	− 0.19	1.19E−07
**y5**	x16	− 0.31	5.96E−19	**y24**	x3	0.32	2.10E−17
**y6**	x2	− 0.08	0.02		x13	− 0.41	9.65E−26
**y7**	x3	− 0.41	1.39E−30	**y25**	x7	− 0.27	5.99E−15
	x13	− 0.66	2.48E−68	**y26**	x2	− 0.48	1.93E−47
**y8**	x16	− 0.29	6.66E−17	**y27**	x2	− 0.41	1.04E−33
**y9**	x15	0.76	1.49E−18	**y28**	x2	− 0.32	1.59E−20
	x17	− 0.19	5.71E−05	**y29**	x15	− 0.35	4.09E−24
	x19	− 0.29	7.53E−05	**y30**	x3	0.71	5.76E-12
**y10**	x15	− 0.69	3.31E−20		x8	− 0.73	1.50E−12
	x19	− 0.49	5.42E−11		x15	0.20	3.63E− 09
**y11**	x2	− 0.31	6.18E−21	**y31**	x15	0.83	1.22E−29
	x3	− 0.89	3.50E−16		x19	− 0.55	1.31E−14
	x8	0.57	4.82E−09	**y32**	x15	− 0.57	7.20E−14
	x11	0.33	2.61E−09		x16	0.16	3.54E−06
**y12**	x13	0.51	9.24E−40		x19	0.25	7.44E−04
	x18	− 0.36	2.26E−21	**y33**	x2	− 0.44	5.19E−17
**y13**	x15	0.23	2.83E−11		x15	0.46	3.37E−18
**y14**	x15	− 0.69	4.85E−20	**y34**	x15	− 0.60	2.53E−15
	x19	0.52	2.41E−12		x19	0.54	1.33E−12
**y15**	x3	− 0.94	2.70E−19	**y35**	x13	− 0.18	1.51E−06
	x8	0.91	3.78E−18		x15	0.75	6.78E−27
**y16**	x1	− 0.51	3.31E−47		x19	− 0.39	1.84E−07
	x3	0.94	1.11E−22	**y36**	x1	0.33	3.05E−20
	x8	− 0.70	6.40E−14		x3	− 0.11	3.50E−25
**y17**	x2	− 0.20	2.02E−08		x8	0.88	7.88E−19
y18	x14	0.03	0.36	**y37**	x15	− 0.17	2.57E−06
**y19**	x15	0.60	4.08E−15	**y38**	x15	0.93	1.73E−36
	x19	− 0.57	5.85E−14		x19	− 0.69	1.38E−21
				**y39**	x16	0.19	4.24E−08

Significant modules are in bold.

**Table 4:** The result of pathway enrichment tests in detected epigenetic subnetworks.

Reference set	GO	Biocarta	CP	KEGG	Reactome
The ratio of enriched subnetworks	1	0.718	1	0.897	1
The ratio of reference sets enriched for subnetworks	0.423	0.498	0.509	0.419	0.435

For each response module, the best subset of predictors was selected based on BIC. We assessed the significance level of regression coefficients in each detected subnetwork to evaluate whether the slope of the regression line differs significantly from zero. Table 3 shows the detailed regression coefficient and the corresponding significance level of each model. Except for the response module *y*18 and *y*21, all models show a significant relationship between the predictor and the response. Thus, we removed the two subnetworks and finally 37 epigenetic subnetworks were kept.

We calculated the confidence score (Table 3) of each selected predictor $x_i$ for response $y_j$, which measures the proportion of variance explained by $x_i$ and the confidence in being a true regulation.

### Follow up analysis

#### Pathway enrichment test and network analysis

To determine the biological functional relevance of the detected epigenetic subnetworks, we performed the pathway enrichment test using reference pathways downloaded from MSigDB^34^, including KEGG^35^, Reactome^36^, Biocarta^37^, GO^38^ and Canonical pathways (CP). The subnetwork is considered to be enriched in a reference pathway if a p-value < 0.05 is obtained by Hypergeometric test after correction. First, we examined the functional homogeneity of the detected subnetworks. A set of genes is defined as functional homogeneity if they are enriched in at least one GO category^38^. We found that all detected subnetworks exhibit significant functional homogeneity since they are all enriched in at least one reference set in GO. Table 4 shows the ratio of enriched subnetworks in each database. All detected subnetworks are enriched in at least one reference pathway from Reactome and CP, and 35 out of 39 subnetworks (90%) and 28 out of 39 subnetworks (72%) were enriched in KEGG and Reactome pathways, respectively. In addition, we evaluated the proportion of reference sets enriched for epigenetic subnetworks (Table 4) and found that 42.3%, 41.9%, 50.9%, 43.5% and 49.8% of reference sets in GO, KEGG, CP, Reactome and Biocarta were enriched for detected subnetworks, respectively. The results revealed that the detected epigenetic subnetworks are of great biological relevance.

Next we asked if the detected subnetworks were related to cancer, especially the breast cancer. We examined whether the genes in detected epigenetic subnetworks are cancer-related biomarkers. We collected 2027 cancer genes from allOnco database (http://www.bushmanlab.org/links/genelists), and 738 breast cancer driver genes from intogen^39^ and OncoSearch^40^. On average, 20% of genes in the detected subnetworks were cancer genes and 9% were breast cancer genes. Table 2 shows the breast cancer genes in detected epigenetic subnetworks, where the third column ’ratio’ represents the ratio between the number of cancer genes and the module size. We found that, except for subnetwork 4, there is at least one breast cancer gene in each detected subnetwork, which reveals that the epigenetic subnetworks are related to breast cancer. In addition, multiple important breast cancer genes were detected in the epigenetic subnetworks, like gene ERBB2 in subnetwork 19, a known proto-oncogene, that encodes HER2, a member of the human epidermal growth factor receptor. Genes TP53BP1 and TP53BP2 were also detected and encode a member of the ASPP (apoptosis-stimulating protein of p53) family of tumor suppressor p53 interacting proteins.

We took the epigenetic subnetwork 16 as an example and performed an extensive analysis for it. The subnetwork 16 contained 20 cancer genes and 9 breast cancer genes (CDK2, PRLR, CDH1, ERBB3, TP53BP2, SRC, MBIP, KDM1A and SERPINE1) and it was enriched in 12 KEGG pathways including two pathways that are specific to the breast cancer: KEGG cell cycle and KEGG P53 signalling pathway. Figure 5 shows the network representation of subnetwork 16, including genes involved in KEGG pathways and genes showing correlations larger than 0.3. Genes acting as predictors were drawn as circles and responses were drawn as squares. Multiple epigenetic mechanisms were detected between predictors and responses. We found that the mechanism between SFN and CDK2 in subnetwork 16 was supported by observations that SFN is a frequently hypermethylated gene^41,42^ emerging as a new inhibitor of CDK2 in breast cancer cells^43^. In addition, SFN has an important function in preventing breast tumor cell growth^43^ which suggests that SFN may play a therapeutic potential role in cancer prevention by targeting epigenetic machinery. We also observed that CCNA1 has been detected as an epigenetic regulator in Fig. 5. Evidence in the literature showed that the differential methylation pattern of CCNA1 was associated with the treatment response in breast cancer and could potentially be a predictive marker to anthracycline/mitomycine sensitivity^44^. Moreover, multiple researches demonstrated that UHRF1 interacting with various proteins in multiple pathways results in the silencing of key tumor suppressor genes in breast cancer^34,45^. In Fig. 5, we observed that the methylation pattern of UHRF1 was highly correlated with the expression of multiple breast cancer genes including ERBB3, TP53BP2 and PRLR. In addition, genes like PLCG1 and PTPN6 in subnetwork 16 were also likely to be epigenetic regulators, which was supported by several researches^46,47^. Overall, these findings supported the idea that our method successfully detects epigenetic subnetworks containing verified epigenetic mechanism, and the detected subnetworks could be a starting point to uncover the underlying epigenetic mechanisms.

#### Survival analysis

**Figure 6:**
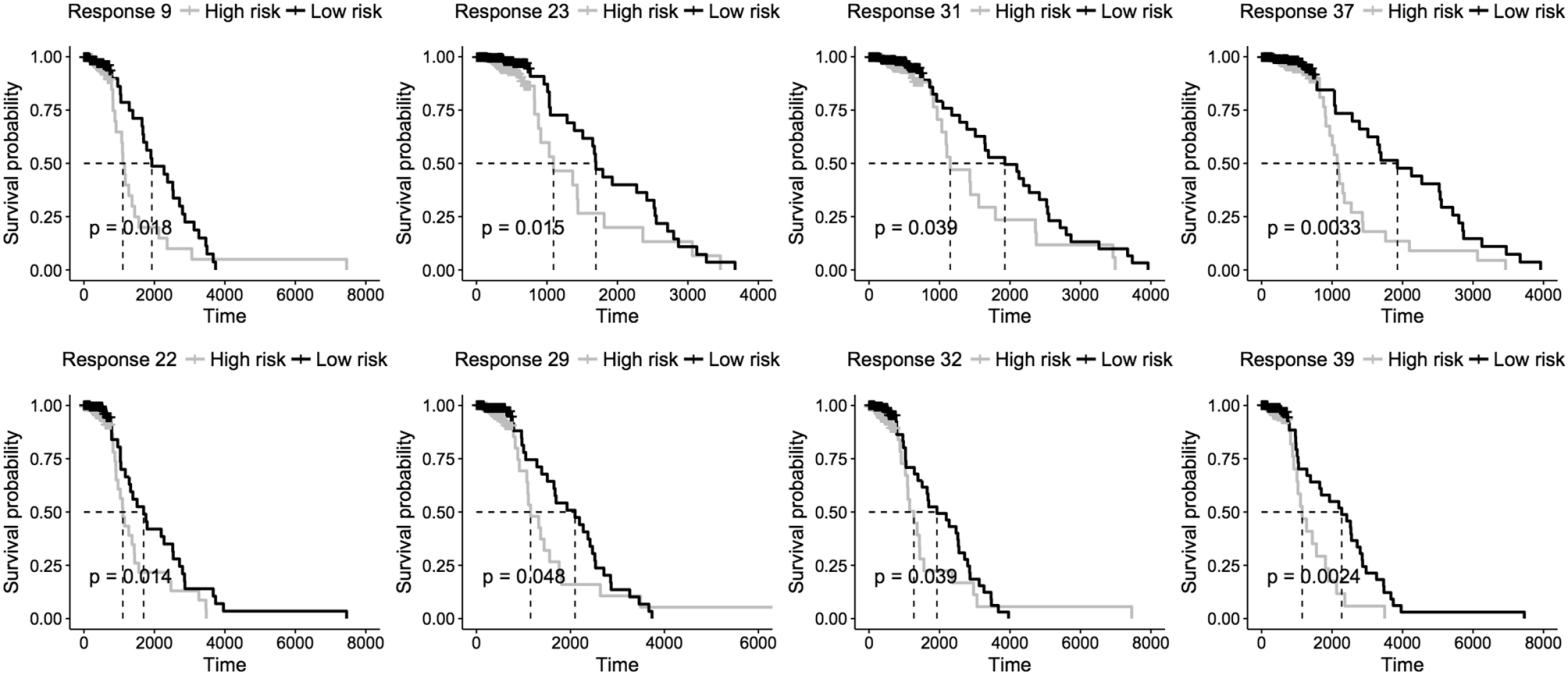
Kaplan–Meier survival analysis for patients in response modules.

**Figure 7:**
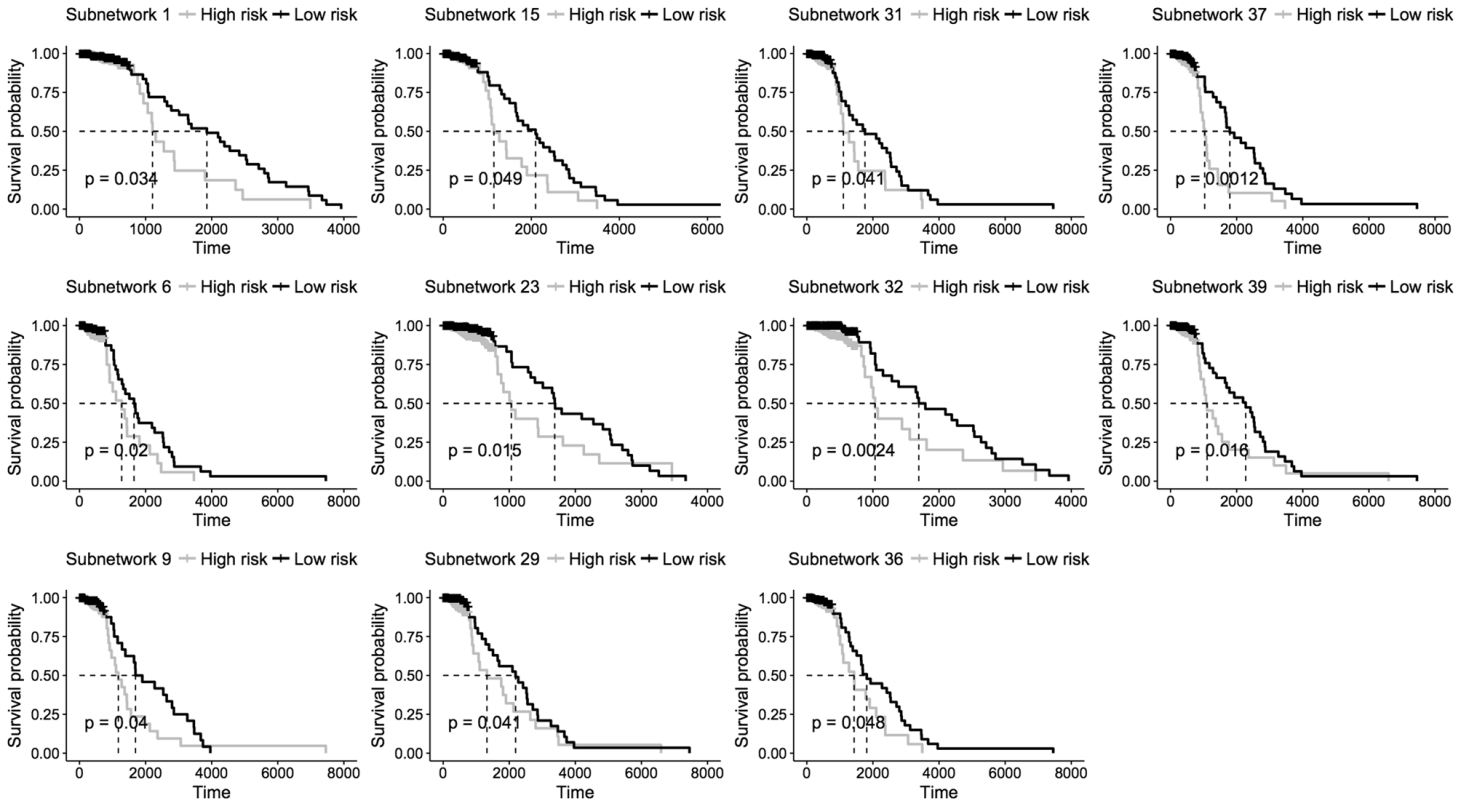
Kaplan–Meier survival analysis for patients in epigenetic subnetworks.

We hypothesized that the profiles of gene expression or DNA methylation in detected modules and subnetworks might be effective prognostic parameters associated with survival time. As introduced in Method, we derived the prognostic index score for each patient based on the module profiles. The patients were divided into high-risk and low-risk groups and we performed the log-rank test to validate if the survival times in the two groups are significantly different. First, the survival analysis was performed on predictor and response modules. The results showed that 8 of 39 response modules (Fig. 6) can divide patients into two groups in which the survival time of patients of high-risk and low-risk are significantly different. However, no groups in predictor modules showed significantly different survival time. Next the multivariate Cox proportional regression was performed on epigenetic subnetworks and we detected that 11 of 37 subnetworks (Fig. 7) were significantly associated with survival time. In addition to the detected 8 significant response modules, 3 more responses in the subnetworks with the incorporation of DNA methylation predictors (subnetworks 1, 6, 36) showed the significant association with survival time, which indicated that the combinations of DNA methylation predictors and responses in the 3 subnetworks improve the classification of patients. It revealed that predictors and response in these 3 subnetworks jointly impact on the survival time.

#### Performance comparison

Ma et al.^16^ detected 26 epigenetic modules by EMDN using TCGA breast cancer data and calculated the ratio of enriched modules as well as the ratio of enriched reference pathways. The method EMDN was compared with two other methods, EpiMod and FEM. They showed that the results detected by EMDN are more enriched than those achieved by EpiMod^14^ and FEM^15^. Since the breast samples used in EMDN, FEM, EpiMod^16^ were identical to the data in our paper, we can compare the performance of epigenetic subnetworks detected by EMDN directly. About 40% to 50% of subnetworks detected by EMDN, EpiMod and FEM were enriched in at least one reference set in GO, KEGG, CP, Reactome and Biocarta, which is much lower than the ratios achieved by our method. In our method, all of subnetworks detected were enriched in GO, CP and Biocarta, and 89.7% and 79.8% of subnetworks were enriched in KEGG and Reactome, respectively. However, one should note that EMDN did not take protein-interactions into account while EpiMod and FEM employed the PPI network, thus we conclude that incorporation with the biological interaction network may contribute to the discovery of biologically-relevant epigenetic subnetworks. The comparison with EMDN revealed that our framework with incorporation with PPI networks can detect more enriched subnetworks than EMDN, EpiMod and FEM.

## Discussion

Recent technology developments have enabled simultaneous genomic profiling of biological samples on multiple platforms, resulting in genome-wide DNA methylation and gene expression data. However, a systematic analysis between the two types of data for discovering biologically relevant combinatorial patterns is currently lacking. In this chapter, we present a method to evaluate the association between gene expression and DNA methylation at the module level by Bayesian regression with the incorporation of prior gene interaction knowledge. We first identified gene expression responses and DNA methylation predictors on a weighted differential expression and methylation networks respectively. Through a significance test, modules passing a p-value threshold were considered as predictors or responses. Density-based and separability-based measures in the significance test were used to validate if detected modules are densely connected and well separated from others. The results showed that the detected modules are well defined and that genes within a module show homogeneity and separability. Then we considered an eigengene as the representative of module profiles for a large proportion of variance of module profiles. With the incorporation of prior gene interaction networks as g-prior, we performed Bayesian regression to discover the dependent relationship between predictors and responses, i.e., the best subset of predictors for each response was selected. The application in breast cancer data demonstrated superior performance of our method to detect biologically relevant epigenetic subnetworks.

Overall, Our contributions lie in the following aspects:

1. We proposed a novel method to detect epigenetic subnetworks by considering a set of highly correlated genes showing the pattern of differential co-expression/methylation instead of considering a single gene as a predictor or response. By comparing with EMDN, EpiMod and FEM which measure the association between gene expression and DNA methylation at the individual gene level, our detected epigenetic subnetworks were much more enriched in biological processes and signalling pathways, which indicates that evaluating the association between gene expression and DNA methylation at the module level would increase the biological association and shed light on the underlying mechanism. Furthermore, our method achieved a larger ratio of enriched subnetworks than that achieved by EMDN. This higher achievement in enrichment ratio is partially due to the construction of significant differential networks with the incorporation of gene interaction information to reduce false positives. The incorporation of the biological interaction networks may contribute to the discovery of enriched epigenetic subnetworks, however it could filter out important cancer genes which were not included in the prior network. Therefore, it remained to be a trade-off between filtering out false positives and discovering novel cancer mechanisms, which could be a future research direction for investigation.
2. By incorporating the prior biological knowledge as g-prior in a Bayesian regression model, it detected more significantly correlated epigenetic subnetworks than the alternative model without g-prior, which showed that encoding biological network information as g-prior effectively guided the selection of epigenetic subnetworks. It is possible to introduce other sources of prior information, such as the derived regulatory interactions in the literature.
3. The network analysis for the detected epigenetic subnetworks revealed the direct causal mechanisms verified in other scientific papers, which indicated the ability of our method in detecting true epigenetic mechanisms and that the detected epigenetic subnetworks could be a good start to uncover underlying epigenetic mechanisms. Moreover, the survival analysis for detected modules and epigenetic subnetworks indicated that the derived modules might be effective prognostic factors associated with the patients’ survival time.

## Supplementary Information

Supplementary Information.

Supplementary Figures.
