# Supplementary material for: Inference of epigenetic subnetworks by Bayesian regression with the incorporation of prior information
Source: Sci Rep. 2022 Nov 23;12:20224. doi: 10.1038/s41598-022-19879-x (PMC9684215; doi:10.1038/s41598-022-19879-x)
Supplement: Supplementary file 1 — Supplementary Information. [file 41598_2022_19879_MOESM1_ESM.pdf]

	1	2	3	4	5	6	7	8	9	10	11	12	13	14	15	16	17	18	19	20
1	0																			
2	4.93E-92	0.00E+00																		
3	2.31E-07	4.44E-13	0.00E+00																	
4	1.45E-11	1.03E-15	2.78E-03	0.00E+00																
5	1.40E-153	4.27E-147	3.65E-38	1.23E-34	0.00E+00															
6	3.82E-22	8.50E-21	5.16E-03	2.86E-09	8.00E-133	0.00E+00														
7	4.06E-51	8.56E-29	3.90E-09	1.64E-02	4.79E-107	2.73E-34	0.00E+00													
8	1.94E-62	3.88E-60	2.02E-35	2.03E-30	9.53E-10	7.25E-50	1.96E-54	0.00E+00												
9	3.09E-04	8.05E-20	2.15E-03	5.57E-09	2.92E-65	5.65E-10	1.85E-25	7.29E-40	0.00E+00											
10	6.28E-107	3.16E-12	2.20E-27	8.68E-20	1.92E-112	5.61E-77	1.07E-41	1.91E-62	2.91E-57	0.00E+00										
11	6.84E-113	7.46E-65	5.23E-13	2.87E-17	3.44E-88	9.21E-51	4.91E-45	1.28E-49	8.58E-49	7.43E-64	0.00E+00									
12	5.71E-48	1.50E-56	3.65E-06	4.87E-02	6.57E-86	1.33E-34	4.41E-11	1.57E-55	7.28E-26	1.43E-73	1.99E-26	0.00E+00								
13	5.47E-73	1.25E-135	3.28E-21	8.27E-05	1.09E-178	8.88E-87	1.13E-11	1.38E-60	1.24E-46	1.46E-108	1.66E-86	4.20E-22	0.00E+00							
14	4.27E-107	4.30E-93	7.35E-40	3.16E-29	6.87E-09	4.92E-111	2.45E-69	1.30E-08	3.82E-35	1.61E-80	6.09E-75	6.65E-92	4.98E-98	0.00E+00						
15	2.68E-40	1.68E-138	3.92E-13	3.44E-30	9.00E-114	4.39E-17	1.87E-104	1.62E-38	5.54E-38	1.84E-125	1.39E-80	4.52E-78	1.99E-137	1.16E-110	0.00E+00					
16	1.21E-49	2.43E-23	2.16E-08	1.13E-03	6.82E-82	1.56E-25	4.22E-02	1.15E-44	7.38E-24	6.52E-43	1.52E-20	4.46E-11	2.92E-27	2.73E-79	4.77E-81	0.00E+00				
17	1.53E-15	1.71E-28	9.82E-06	8.82E-04	2.51E-84	4.36E-13	1.15E-08	4.92E-44	4.45E-12	5.42E-57	6.50E-59	4.56E-16	3.73E-15	9.36E-56	3.09E-69	4.49E-11	0.00E+00			
18	4.85E-15	2.17E-14	8.20E-11	7.12E-10	7.35E-14	6.88E-13	1.59E-14	1.79E-10	1.35E-13	4.11E-14	1.00E-10	4.38E-15	1.18E-13	3.61E-11	2.45E-10	2.21E-12	5.02E-13	0.00E+00		
19	2.53E-20	2.93E-55	6.53E-03	8.66E-08	5.48E-134	2.10E-16	8.09E-35	4.61E-48	2.77E-06	1.66E-77	8.03E-43	2.15E-15	1.23E-73	1.26E-65	6.56E-34	2.21E-26	2.05E-17	7.23E-11	0.00E+00	
20	1.45E-91	3.18E-133	2.71E-11	2.94E-03	1.62E-212	2.91E-40	7.22E-31	9.00E-70	1.98E-47	1.93E-142	1.68E-33	4.61E-26	1.79E-65	3.07E-139	8.32E-137	2.34E-15	1.31E-19	4.30E-15	3.11E-58	0.00E+00
21	4.44E-63	5.37E-62	1.42E-27	2.36E-21	5.27E-58	5.72E-52	4.02E-63	1.46E-37	2.35E-42	5.48E-62	1.59E-53	5.48E-54	2.38E-83	8.53E-60	1.46E-64	2.81E-44	3.91E-60	2.70E-12	1.42E-53	1.07E-105
22	1.47E-06	3.17E-111	2.23E-17	3.26E-14	3.74E-131	6.80E-49	1.48E-66	6.56E-64	6.89E-11	7.15E-125	3.20E-102	4.80E-52	1.53E-63	1.46E-96	2.72E-74	1.10E-53	2.99E-22	2.00E-15	5.41E-28	2.92E-130
23	1.92E-78	1.12E-91	7.39E-35	1.12E-23	7.67E-54	1.02E-70	1.19E-71	1.18E-33	2.67E-58	1.15E-58	2.27E-67	7.92E-74	1.32E-91	7.45E-45	2.48E-33	6.96E-51	6.33E-84	1.16E-11	1.97E-56	1.11E-128
24	2.63E-75	2.04E-07	2.18E-09	4.08E-07	3.59E-75	3.58E-18	4.54E-11	9.34E-51	2.24E-36	2.26E-20	4.00E-11	4.34E-17	1.68E-44	6.46E-90	4.51E-85	2.33E-07	2.94E-22	6.42E-14	1.37E-28	2.66E-34
25	8.68E-20	8.04E-12	6.16E-07	6.05E-05	1.47E-38	7.97E-14	3.91E-04	3.08E-17	3.70E-09	2.85E-24	1.62E-16	1.09E-06	1.61E-07	4.88E-17	4.84E-44	5.15E-05	4.33E-07	5.43E-11	8.70E-15	4.83E-07
26	3.31E-43	8.40E-116	6.07E-06	5.80E-03	2.28E-162	3.91E-52	5.58E-15	1.60E-56	8.63E-18	3.19E-100	2.38E-39	5.39E-06	2.19E-21	1.22E-120	2.98E-115	2.43E-10	6.60E-23	5.63E-15	1.00E-22	2.89E-45
27	2.02E-83	4.23E-51	2.43E-13	3.45E-04	1.43E-159	1.58E-35	9.33E-09	5.56E-72	1.13E-41	1.40E-65	3.62E-31	4.27E-16	6.99E-42	1.50E-103	6.46E-144	2.28E-07	5.87E-18	2.29E-14	7.83E-46	1.36E-13
28	3.10E-19	6.79E-58	5.47E-07	3.67E-03	2.18E-97	9.31E-32	1.70E-07	7.26E-50	2.15E-07	1.47E-64	9.93E-43	1.14E-04	5.63E-12	7.76E-79	9.25E-90	5.08E-07	7.82E-11	6.29E-15	1.20E-11	1.71E-26
29	6.50E-220	5.07E-192	3.80E-38	5.16E-10	2.95E-168	1.21E-125	4.08E-25	2.77E-74	1.87E-98	1.72E-137	2.44E-46	2.65E-38	1.06E-64	1.40E-135	3.87E-141	4.42E-15	1.10E-67	3.28E-13	6.67E-127	2.11E-78
30	3.59E-203	1.23E-143	4.66E-33	3.28E-10	3.95E-147	1.24E-112	1.04E-15	2.21E-60	5.45E-87	3.49E-91	6.99E-63	1.86E-40	3.22E-54	3.95E-98	3.04E-162	1.84E-11	2.30E-65	8.12E-12	1.24E-98	5.26E-79
31	1.57E-17	3.72E-79	7.29E-07	9.49E-12	4.83E-171	1.04E-15	2.38E-54	3.86E-54	1.36E-15	2.39E-137	7.26E-114	1.28E-42	1.48E-101	9.69E-114	1.19E-31	1.84E-50	3.80E-14	1.77E-12	5.90E-18	4.25E-143
32	2.30E-42	1.16E-110	9.24E-37	1.67E-28	6.73E-86	4.31E-50	1.38E-91	4.08E-42	6.24E-31	2.55E-85	8.68E-76	1.04E-93	4.25E-94	6.19E-85	2.18E-25	7.88E-71	1.64E-55	6.24E-11	1.67E-55	1.60E-147
33	5.00E-23	6.48E-17	2.76E-03	8.61E-06	1.61E-73	2.28E-08	2.14E-23	1.55E-36	2.43E-12	3.37E-31	4.60E-15	3.52E-18	3.61E-67	2.65E-56	3.67E-23	6.36E-20	9.16E-18	3.90E-12	1.33E-08	5.63E-32
34	4.04E-17	1.75E-129	2.71E-09	4.28E-13	3.20E-130	3.28E-28	1.15E-79	1.21E-51	4.43E-19	8.58E-123	6.61E-86	1.60E-30	5.57E-87	2.17E-97	1.32E-20	2.76E-67	5.59E-30	2.29E-12	5.75E-18	4.54E-99
35	3.50E-25	1.36E-75	7.83E-13	1.24E-17	3.41E-118	6.17E-28	1.10E-52	4.51E-56	1.28E-06	1.06E-79	2.39E-70	9.83E-65	1.34E-95	4.07E-92	1.29E-58	1.79E-33	6.85E-29	1.16E-10	3.53E-11	9.10E-142
36	9.77E-151	1.31E-53	1.64E-23	1.90E-08	2.83E-130	7.73E-77	5.96E-16	2.63E-53	9.06E-55	7.80E-45	5.17E-22	4.86E-20	4.98E-71	2.55E-106	1.86E-143	1.22E-07	1.40E-54	5.34E-12	2.33E-70	2.41E-69
37	2.00E-06	9.39E-62	2.63E-11	9.16E-14	1.85E-50	2.75E-18	3.50E-40	6.16E-27	4.74E-11	8.86E-48	4.01E-40	9.76E-37	1.04E-57	1.71E-43	1.68E-04	3.12E-34	6.08E-34	1.38E-09	3.64E-13	3.66E-48
38	1.72E-52	3.15E-15	1.07E-04	4.48E-04	3.79E-117	7.49E-13	3.38E-06	4.74E-60	3.45E-21	2.05E-50	1.14E-34	1.53E-08	2.51E-36	3.29E-105	1.08E-90	1.03E-08	3.30E-08	6.22E-14	5.89E-28	1.01E-13
39	2.12E-79	1.05E-72	2.58E-26	3.87E-21	8.19E-61	8.56E-53	2.02E-51	7.80E-29	2.09E-51	1.82E-50	3.66E-13	3.10E-45	1.45E-73	5.40E-49	2.82E-49	5.32E-35	4.85E-50	8.35E-11	1.39E-57	1.29E-57

21	22	23	24	25	26	27	28	29	30	31	32	33	34	35	36	37	38	39
0.00E+00																		
1.27E-96	0.00E+00																	
1.66E-40	8.47E-84	0.00E+00																
4.99E-47	3.27E-91	3.81E-60	0.00E+00															
1.92E-30	1.05E-20	2.35E-35	6.78E-07	0.00E+00														
1.11E-63	1.35E-43	7.96E-75	2.11E-43	1.65E-07	0.00E+00													
5.18E-72	4.30E-108	1.54E-115	8.55E-05	2.52E-05	1.43E-38	0.00E+00												
1.41E-52	5.52E-17	1.66E-65	2.99E-25	1.06E-05	4.17E-05	3.48E-16	0.00E+00											
3.41E-76	1.27E-206	1.17E-76	1.69E-45	7.51E-15	5.68E-48	6.82E-44	2.43E-36	0.00E+00										
1.60E-82	4.85E-189	1.35E-67	1.15E-24	6.60E-06	3.33E-52	9.77E-29	3.01E-38	6.63E-12	0.00E+00									
5.63E-83	5.56E-17	6.02E-73	4.11E-63	1.21E-28	1.22E-52	9.20E-125	6.85E-32	2.26E-258	1.55E-266	0.00E+00								
1.64E-56	1.30E-36	7.47E-17	1.39E-60	1.44E-34	2.27E-94	1.88E-108	6.41E-66	2.07E-123	5.78E-148	1.43E-35	0.00E+00							
1.06E-41	4.21E-50	5.46E-43	5.81E-13	4.39E-19	4.20E-35	1.71E-18	5.84E-28	1.92E-63	1.68E-60	2.81E-19	4.41E-42	0.00E+00						
4.95E-69	4.21E-20	1.28E-62	4.10E-88	1.68E-26	2.37E-42	6.46E-104	3.81E-29	5.45E-155	1.53E-177	8.48E-17	5.58E-42	3.54E-48	0.00E+00					
3.51E-60	6.91E-22	4.67E-58	9.09E-76	1.42E-26	1.96E-55	2.35E-83	5.47E-26	3.88E-155	9.89E-171	1.92E-35	1.06E-35	5.31E-23	2.24E-65	0.00E+00				
4.39E-69	7.56E-125	4.92E-87	5.38E-07	9.50E-08	1.40E-47	3.50E-16	5.70E-23	1.42E-19	1.96E-13	2.96E-136	8.48E-104	3.58E-28	7.78E-114	9.38E-94	0.00E+00			
2.09E-32	6.77E-13	3.78E-18	1.19E-29	2.70E-26	2.46E-27	1.77E-50	4.94E-27	6.16E-50	1.45E-52	1.71E-09	2.59E-06	8.17E-14	1.37E-08	2.79E-13	1.95E-45	0.00E+00		
5.83E-66	2.44E-70	2.06E-81	2.49E-05	2.10E-06	7.40E-15	3.91E-09	1.21E-13	7.23E-54	3.58E-37	1.75E-58	3.76E-110	3.04E-15	8.77E-55	9.28E-63	2.99E-23	1.06E-50	0.00E+00	
2.05E-35	6.75E-78	2.76E-21	5.23E-24	2.98E-23	1.47E-76	5.25E-45	6.02E-47	4.87E-19	6.83E-22	6.42E-73	3.38E-35	3.83E-44	1.62E-53	7.88E-59	2.85E-18	2.06E-34	2.48E-50	0.00E+00
