# Supplementary material for: Inference of epigenetic subnetworks by Bayesian regression with the incorporation of prior information
Source: Sci Rep. 2022 Nov 23;12:20224. doi: 10.1038/s41598-022-19879-x (PMC9684215; doi:10.1038/s41598-022-19879-x)
Supplement: Supplementary file 2 — Supplementary Figures. [file 41598_2022_19879_MOESM2_ESM.pdf]

Inference of epigenetic subnetworks by Bayesian regression with the incorporation of prior information

Anqi Jing[1,*] and Jie Han[1,*]

[1]Department of Electrical and Computer Engineering, University of Alberta, Edmonton, T6G 1H9, Canada

[*]ajing@ualberta.ca, jie8@ualberta.ca

Supplementary figures

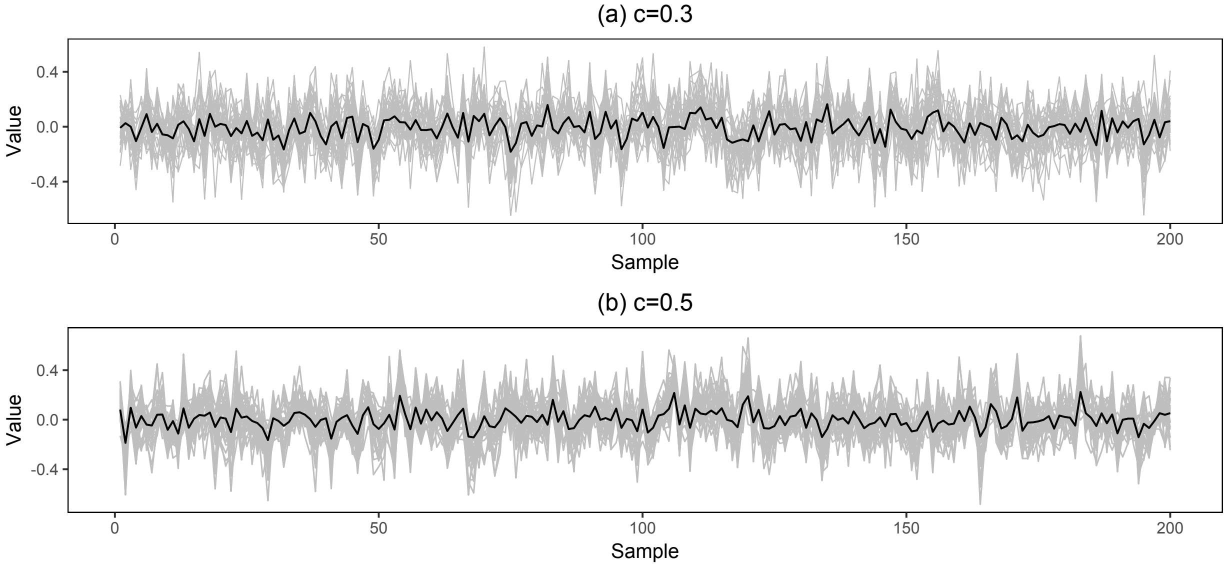

Figure 1. Methylation levels of simulated module genes (grey lines) and eigengene (black line) in 200 samples. (a) The correlation signal within the module is 0.3. (b) The correlation signal is set to 0.5.

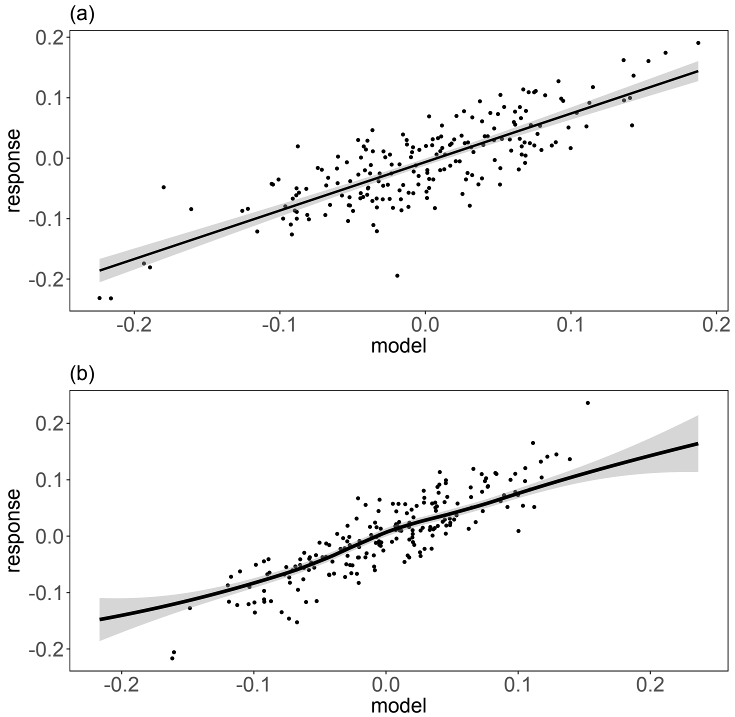

Figure 2. Fitting regression models. (a) The model for subnetworks consisting of response y_2^2 and predictor x_2 with association signal 0.1. (b) The model for subnetworks consisting of response y_3^2 and predictor x_1 with association signal 0.1.

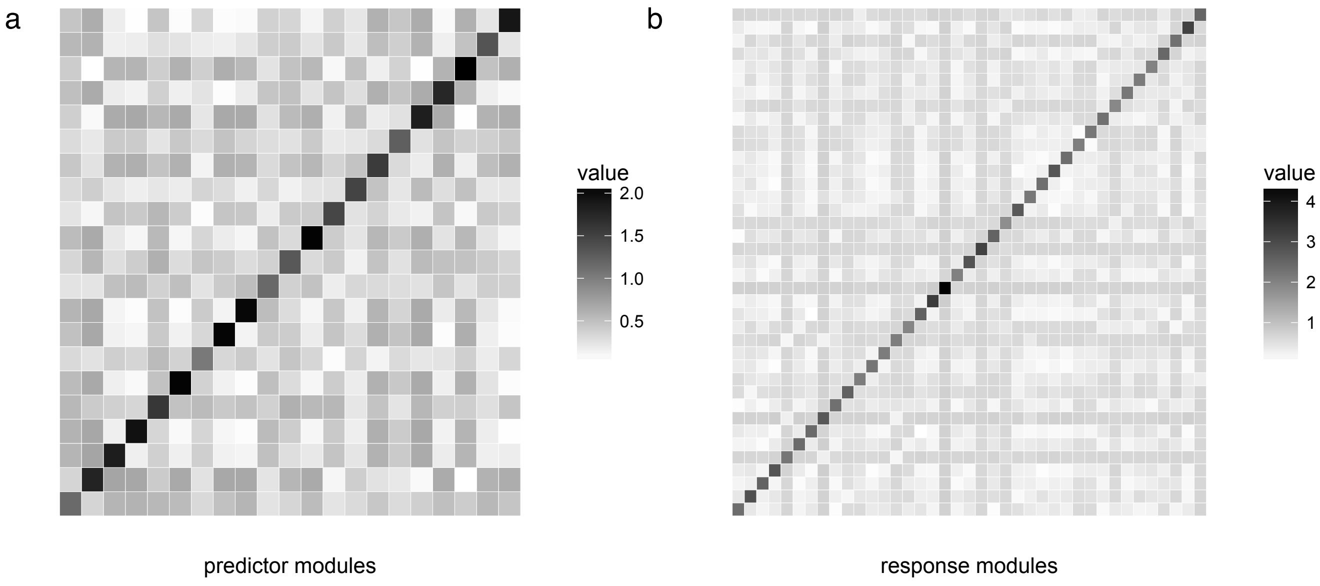

Figure 3. Heatmaps of separability and density scores for predictors (a) and responses (b).

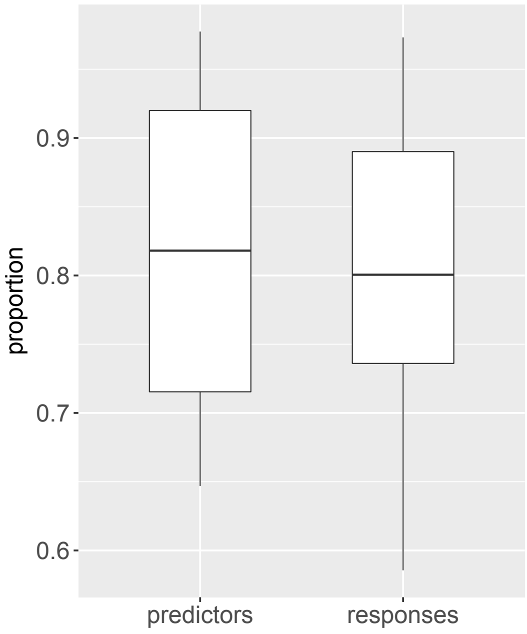

Figure 4. The proportion of variance explained by eigengenes.

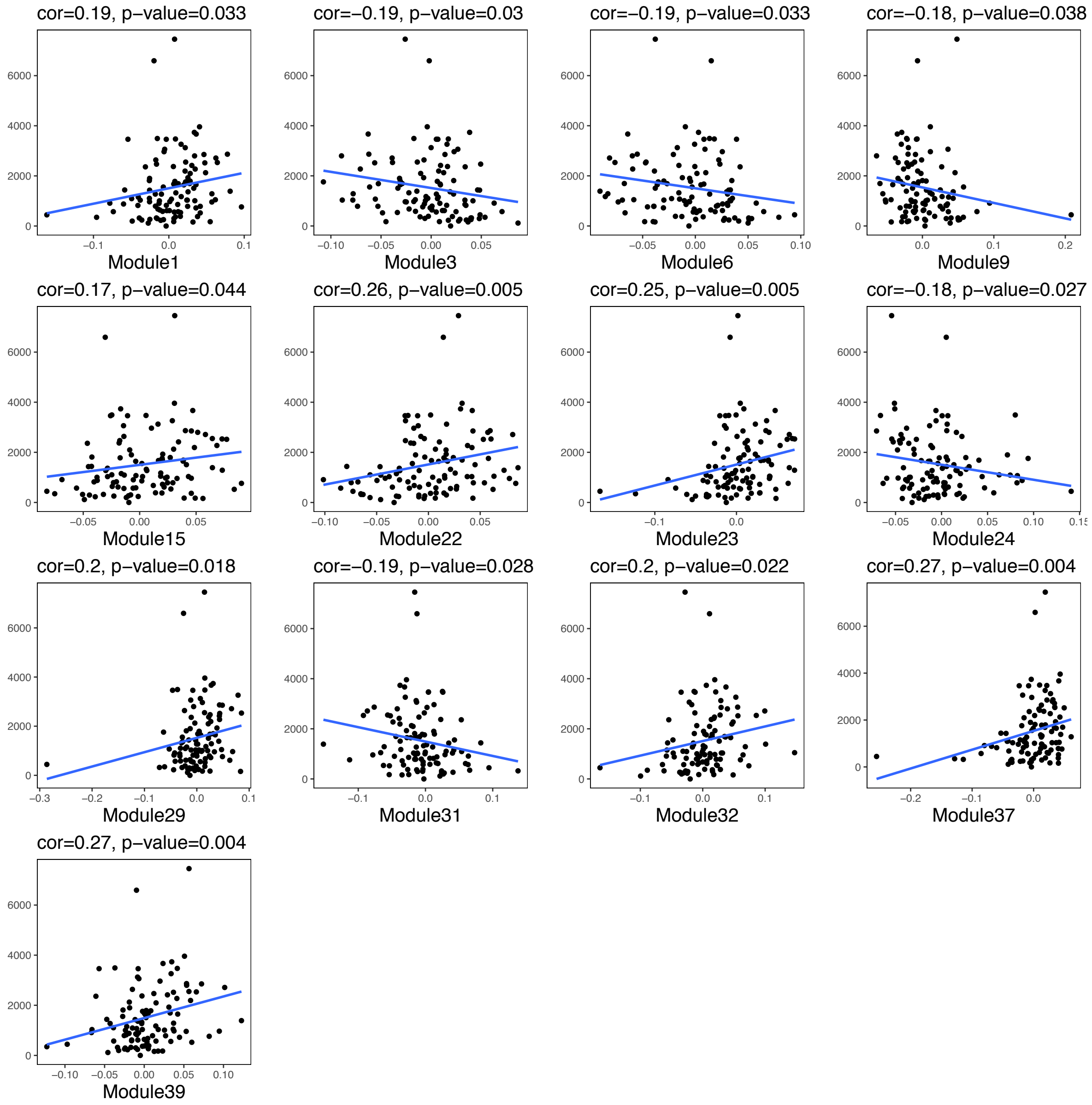

Figure 5. Scatterplots between the eigengenes of response modules and the patient survival time. In each figure, a dot represents a patient, and the x-axis and y-axis represent the profile of the module eigengene and the corresponding patient survival days, respectively.
